# HOF‐Stabilized Apple EV Nanocargo in an NIR‐Responsive Hydrogel Enables On‐Demand Therapeutic Modulation for Transdermal Deep‐Joint Osteoarthritis Therapy

**DOI:** 10.1002/adhm.71330

**Published:** 2026-07-15

**Authors:** Yu‐Wen Tseng, Pei‐Wei Weng, Hsien‐Tsung Lu, Jiunn‐Horng Kang, Lekha Rethi, Lekshmi Rethi, Yan‐Ting Chen, Hieu Trung Nguyen, Yao‐Tung Tsai, Andrew E.‐Y. Chuang

**Affiliations:** ^1^ Graduate Institute of Biomedical Materials and Tissue Engineering International Ph.D. Program in Biomedical Engineering College of Biomedical Engineering Taipei Medical University New Taipei City Taiwan; ^2^ Department of Orthopedics, Shuang Ho Hospital Taipei Medical University New Taipei City Taiwan; ^3^ Department of Orthopedics School of Medicine College of Medicine Taipei Medical University Taipei City Taiwan; ^4^ Research Center of Biomedical Devices Taipei Medical University Taipei Taiwan; ^5^ Graduate Institute of Nanomedicine and Medical Engineering College of Biomedical Engineering Taipei Medical University Taipei Taiwan; ^6^ Department of Orthopedics Taipei Medical University Hospital Taipei Taiwan; ^7^ Department of Orthopedics School of Medicine College of Medicine Taipei Medical University Taipei Taiwan; ^8^ International Ph.D. Program in Cell Therapy and Regenerative Medicine College of Medicine Taipei Medical University Taipei Taiwan; ^9^ Department of Physical Medicine and Rehabilitation Taipei Medical University Hospital Taipei Taiwan; ^10^ Department of Physical Medicine and Rehabilitation School of Medicine College of Medicine Taipei Medical University Taipei Taiwan; ^11^ Department of Orthopedics and Trauma Faculty of Medicine University of Medicine and Pharmacy at Ho Chi Minh City Ho Chi Minh City Vietnam; ^12^ Department of Orthopedic Surgery Tri‐Service General Hospital National Defense Medical University Taipei Taiwan; ^13^ Cell Physiology and Molecular Image Research Center Taipei Medical University‐Wan Fang Hospital Taipei Taiwan; ^14^ Precision Medicine and Translational Cancer Research Center Taipei Medical University Hospital Taipei Taiwan

**Keywords:** hydrogel, immunological dysregulation, nanomedicine, osteoarthritis (OA), OA‐driven pathogenesis modulation, oxidative imbalance/stress

## Abstract

Osteoarthritis (OA) is driven by intertwined pathological processes, including chronic inflammation, oxidative stress, immune imbalance, hypoxia, and aberrant angiogenesis, while current noninvasive therapies remain limited by insufficient deep‐joint delivery and poor multidimensional regulation. Here, an extracellular vesicle (EV)‐centered, near‐infrared (NIR)‐responsive transdermal platform is developed to address these challenges. Apple‐derived extracellular vesicles (AEVs) are used as intrinsically bioactive nanocarriers to co‐deliver berberine (BBR) and piperine (PIP), and are integrated into a biocompatible dextran/alginate hydrogel containing graphene oxide (GO). Under NIR stimulation, GO provides on‐demand energy conversion that supports mild hyperthermia‐assisted barrier loosening together with potential photo‐redox‐associated microenvironment modulation, thereby enhancing local therapeutic availability while maintaining a safe thermal window. In vivo assessment of TRPV1 expression and tight‐junction‐related responses provides supportive indication for transport‐associated modulation, whereas ex vivo diffusion findings are interpreted as barrier‐level permeation evidence rather than direct confirmation of in vivo mechanisms. In an OA rat model, the NIR‐activated platform is associated with reduced joint inflammation and edema, attenuated oxidative stress, improved macrophage polarization toward an anti‐inflammatory phenotype, enhanced gait‐related joint function, and preservation of cartilage structure. This study establishes a noninvasive, stimulus‐responsive transdermal strategy for localized and multifaceted OA microenvironment modulation.

## Graphical abstract

An NIR‐responsive transdermal hydrogel integrating apple EV nanocargo, berberine, piperine, HOFs, and graphene oxide enables localized osteoarthritis modulation. The platform supports enhanced transdermal delivery together with mild hyperthermia‐associated and redox‐related responses, leading to reduced inflammation and oxidative stress, improved macrophage polarization, and preservation of cartilage structure in vivo.

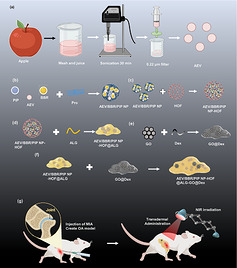

## 1 Introduction

Osteoarthritis (OA) is a prevalent and progressive joint disorder that causes chronic pain, reduced mobility, and substantial functional limitations, largely driven by cartilage degeneration and persistent synovial inflammation [1, 2]. At the molecular level, excessive reactive oxygen species (ROS) such as hydroxyl radicals, superoxide anions, and hydrogen peroxide contribute to OA progression by inducing oxidative stress, DNA damage, lipid peroxidation, and cell death [3, 4, 5]. These processes amplify proinflammatory signaling and disrupt the balance between pro‐ and anti‐inflammatory mediators, thereby contributing to progressive cartilage degeneration and impaired joint function [6]. Meanwhile, hypoxia within the OA microenvironment further exacerbates inflammation by upregulating hypoxia‐inducible factor (HIF)‐1α, aggravating tissue injury and impairing immune regulation [7, 8]. Oxidative stress and hypoxia together bias macrophage polarization toward a proinflammatory M1 phenotype and intensify inflammatory cascades through pathways such as nuclear factor (NF)‐κB [9, 10]. Accordingly, strategies aimed at promoting macrophage polarization toward an anti‐inflammatory M2 phenotype are increasingly viewed as a potentially beneficial approach for mitigating inflammation and preserving cartilage homeostasis [11]. Beyond local inflammation, the bone immune microenvironment (BIM) is increasingly recognized as a regenerative regulator; M2 polarization can enhance macrophage–mesenchymal stem cell (MSC) crosstalk and favor tissue repair and bone healing [12, 13, 14]. Aberrant angiogenesis represents another OA hallmark; elevated vascular endothelial growth factor (VEGF) contributes to pathological neovascularization and pain, and VEGF inhibition has been associated with slowed OA progression [15].

Despite growing insights into OA pathophysiology, current therapeutic options remain limited by inadequate target‐site delivery and concerns over long‐term safety and tolerability. Oral therapies often suffer from low bioavailability and systemic side effects, while intra‐articular injections provide localized benefit but introduce procedure‐related risks and burdens due to repeated administration. These limitations motivate noninvasive strategies that can deliver therapeutics beyond the skin barrier and simultaneously modulate the intertwined OA microenvironment, including inflammation, oxidative stress, hypoxia, and angiogenesis.

Here, we developed a multifunctional, near‐infrared (NIR)‐responsive transdermal hydrogel system, termed AEV/BBR/PIP NP‐HOF@ALG‐GO@Dex, designed to integrate complementary bioactive modules within a programmable platform. The formulation integrates *Malus domestica* (apple)‐derived extracellular vesicles (AEVs) as natural nanocarriers with reported anti‐inflammatory and antioxidant bioactivity, berberine (BBR) as a functional component associated with antioxidant and anti‐angiogenic effects, and piperine (PIP), which has been implicated in barrier‐associated and immunoregulatory pathways linked to TRPV1 in living tissues [16, 17]. Graphene oxide (GO) was incorporated as an NIR‐responsive component with potential photothermal and electroactive properties to provide on‐demand local stimulation and a physicochemical context potentially relevant to redox‐associated microenvironmental modulation [18, 19, 20]. To enhance cargo programmability and retention, hydrogen‐bonded organic frameworks (HOFs) were integrated as porous crystalline assemblies to improve encapsulation efficiency and support formulation stability and bioavailability‐associated behavior. The entire construct was embedded in a biocompatible alginate–dextran (ALG–Dex) hydrogel matrix to ensure structural stability, conformal skin contact, and sustained local availability for transdermal delivery.

A central design objective of this work was to move beyond a purely heat‐driven transdermal paradigm. Under NIR stimulation, the GO‐containing hydrogel was designed to provide on‐demand, stimuli‐responsive effects, whereby mild hyperthermia may transiently reduce barrier resistance and enhance local transport, while redox‐associated changes may provide a supportive physicochemical context relevant to the inflammatory and oxidative microenvironment. Within a living OA context, we evaluated whether this combined stimulation improves deep‐joint therapeutic engagement while maintaining a safe thermal window. In parallel, the AEV‐centered nanocargo design was expected to contribute intrinsic bioactivity and improve local tissue interactions, whereas the inclusion of BBR and PIP was designed to broaden the therapeutic bandwidth toward oxidative stress reduction, angiogenesis suppression, and immune rebalancing.

In contrast to prior GO‐based photothermal hydrogels and NIR‐assisted transdermal systems that largely emphasize heat generation and generic permeation enhancement, our platform was built around an EV‐centered translational premise: plant‐derived EVs serve as a dual‐function therapeutic and delivery module for noninvasive deep‐joint intervention. Building on this premise, HOF‐stabilized nanocargo engineering provided an additional level of structural programmability beyond simple drug‐in‐gel blending, while GO‐enabled energy conversion introduced stimulus‐responsive, multi‐output regulation. Together, the resulting system was developed to support joint‐level therapeutic modulation in OA beyond simple enhancement of transdermal transport.

## 2 Results and Discussion

### 2.1 Physicochemical Profiling and Functional Assessment of *Malus domestica (M. domestica)* (Apple)‐Derived Extracellular Vesicles (AEVs)

**FIGURE 1:**
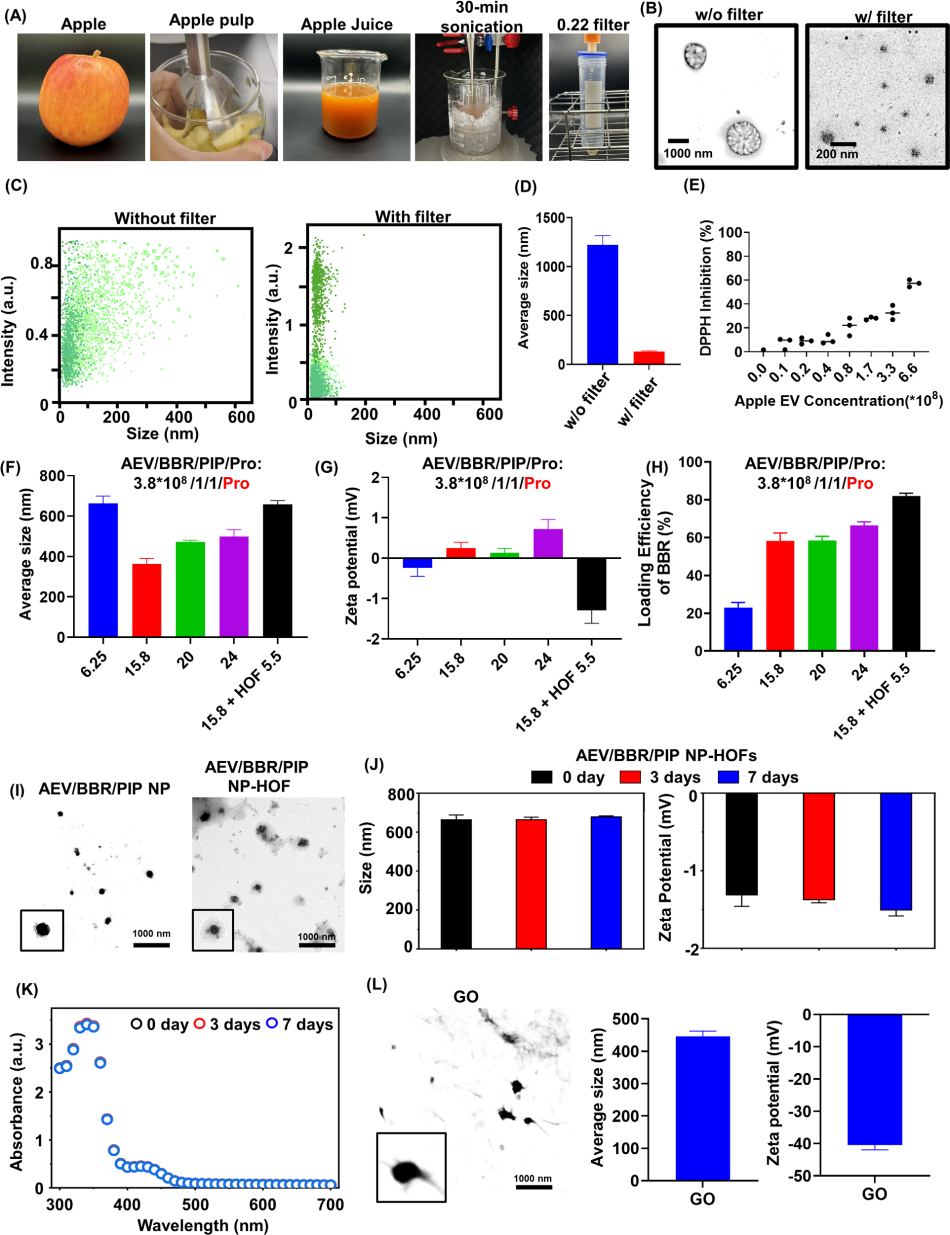
Preparation, characterization, and functional evaluation of *Malus domestica* (apple)‐derived extracellular vesicles (AEVs). (A) Photographic depiction of the AEV preparation process, including *M. domestica* (apple) homogenization, extraction, differential centrifugation, and filtration. (B) Transmission electron microscopic (TEM) images reveal spherical vesicles in filtered AEVs (∼100 nm) and debris in unfiltered samples. Scale bars: 1000 nm (left TEM), 200 nm (right TEM). (C) A nanoparticle tracking analysis (NTA) comparing size distributions of filtered and unfiltered AEVs, showing improved homogeneity (50–150 nm) in filtered samples. (D) Dynamic light scattering (DLS) analysis demonstrating a narrower size distribution and lower variation for filtered AEVs. (E) DPPH inhibition assay showing that AEVs possessed antioxidant ability. (F) DLS analysis of particle sizes with various protamine (Pro) and hydrogen‐bonded organic framework (HOF) concentrations, showing the smallest size (∼362 nm) at 15.8 mg/mL Pro, with HOFs stabilizing the size at ∼658 nm. (G) Zeta potential measurements, demonstrating increasing cationic charges with higher Pro concentrations and charge neutralization upon HOF inclusion. (H) Loading efficiency of berberine (BBR), with a peak of ∼58.14% at 15.8 mg/mL Pro and further enhancement to ∼81.8% with HOFs. (I) TEM images showing the spherical morphology of AEV/BBR/PIP NPs, the relatively uniform core–shell–like structures of AEV/BBR/PIP NP‐HOFs. (J) DLS‐based stability analysis of AEV/BBR/PIP NP‐HOFs at days 0, 3, and 7, showing no obvious change in particle size and indicating good colloidal stability over time. (K) UV–vis absorption spectra of AEV/BBR/PIP NP‐HOFs at different time points, showing no significant spectral shift or intensity change, suggesting preserved structural and compositional stability. (L) TEM images and DLS analysis of GO, and the planar sheet‐like morphology of graphene oxide (GO), consistent with the distinct structural characteristics of each component.

Figure 1 showcases the preparation, characterization, and functional evaluation of AEVs. As shown in the preparation process (Figure 1A), the photographic data involved a series of steps, starting with *M. domestica* homogenization and extraction, followed by differential sonication and filtration. TEM images (Figure 1B) revealed distinct morphological differences between filtered and unfiltered samples for assaying AEV structural changes. Unfiltered samples contained aggregates and debris, while filtered samples displayed well‐defined, spherical vesicles with sizes of around 100 nm. This supports the effectiveness of filtration in purifying AEVs for downstream applications. As shown in Figure 1C, NTA data were used to compare AEVs with and without filtration. Filtered AEVs exhibited a more uniform particle size distribution, predominantly ranging 50–150 nm, while unfiltered samples showed a broader size variability. Filtration therefore improved the homogeneity of the AEV preparation, which is beneficial for formulation reproducibility and subsequent biological assessment. The DLS analysis (Figure 1D) aligned with the NTA and TEM findings, showing a narrower size distribution and lower variation for filtered AEVs compared to unfiltered ones. This uniformity is essential for predictable pharmacokinetics and biodistributions in therapeutic settings. The antioxidative potential of AEVs was evaluated via DPPH inhibition (Figure 1E). AEVs exhibited a concentration‐dependent DPPH‐inhibition capacity, consistent with antioxidant‐associated activity [21].

### 2.2 Optimization and Structural Analysis of AEV/BBR/PIP NP Formulations with Pro and HOFs

Figure 1 presents the characterization of AEV/BBR/PIP NPs and their formulations with Pro and HOFs, along with the corresponding optimization of the formulation conditions. As shown in Figure 1F, DLS data revealed particle size distributions of AEV/BBR/PIP NPs formulated with various Pro and HOF concentrations. At 6.25 mg Pro, the particle size was approximately 662.9 nm. Increasing the Pro concentration resulted in a significant reduction in the particle size, with the smallest size (∼362.8 nm) observed at 15.8 mg/mL Pro. However, further increasing Pro to 24 mg/mL led to particle aggregation and a size increase (∼499 nm). Therefore, the formulation containing 15.8 mg/mL Pro, which achieved the smallest particle size, was selected for further applications. The incorporation of HOFs (15.8 mg/mL Pro and 5.5 mg/mL HOFs) yielded particles with an average size of approximately 600 nm.

Figure 1G presents zeta potential data, which demonstrate surface charge dynamics of the formulations. Higher Pro concentrations enhanced the cationic charge, with zeta potential peaking at ∼+0.722 mV with 24 mg/mL Pro. The addition of HOFs resulted in a reduction (∼−1.29mV), likely due to charge neutralization by HOF components, which is favorable for cellular uptake and systemic stability. The loading efficiency (LE) with BBR (Figure 1H) progressively increased with rising Pro concentrations, achieving approximately 58.1% at 15.8 mg/mL Pro. HOF incorporation further boosted the LE to around81.8%, suggesting the role in enhancing the drug‐loading capacity by providing a robust and stable matrix for bioactive molecules.

TEM images (Figure 1I) further substantiated these findings, revealing distinct morphological variations among AEV/BBR/PIP NPs, AEV/BBR/PIP NP‐HOF exhibited a spherical morphology with notable size heterogeneity. HOF incorporation transformed the NPs into well‐defined, core–shell structures, indicating a structural consistency. The data further support the physicochemical stability of the HOF‐containing formulation under the tested conditions. Specifically, stability was assessed at Day 0, 3, and 7 using DLS size/zeta‐potential measurements (Figure 1J) and absorption spectral analysis (Figure 1K). The results showed no obvious changes in particle characteristics or spectral features throughout the 7‐day testing period, supporting the stability of the HOF‐containing nanoplatform under the examined conditions.

As GO was used as an additional structural component in the subsequent hydrogel formulation together with AEV/BBR/PIP NP‐HOFs, its physicochemical characteristics were also examined. TEM, DLS, and zeta potential analyses of GO (Figure 1L) revealed its characteristic sheet‐like morphology, a particle size of ∼446 nm, and a zeta potential of −40 mV, consistent with its distinct physicochemical properties. Supplementary characterization further demonstrated the NIR‐responsive functionality of GO. Under cyclic NIR irradiation, GO in the water system generated a measurable photo‐response (Figure S1A), indicating its photoresponsive behavior. In parallel, GO also exhibited photothermal conversion under NIR exposure (Figure S1B). Furthermore, dissolved oxygen measurements showed detectable changes in dissolved oxygen levels in the GO‐containing water system during NIR irradiation (Figure S1C). Under NIR irradiation, the GO‐containing formulation showed altered oxygen‐related behavior, including an increase in dissolved oxygen under the tested conditions. The NMR data indicated the chemical shift of formulations (Figure S1D).

### 2.3 Photothermal‐Responsive Behavior and Functional Characterization of AEV/BBR/PIP NP‐HOF@ALG‐GO@Dex

**FIGURE 2:**
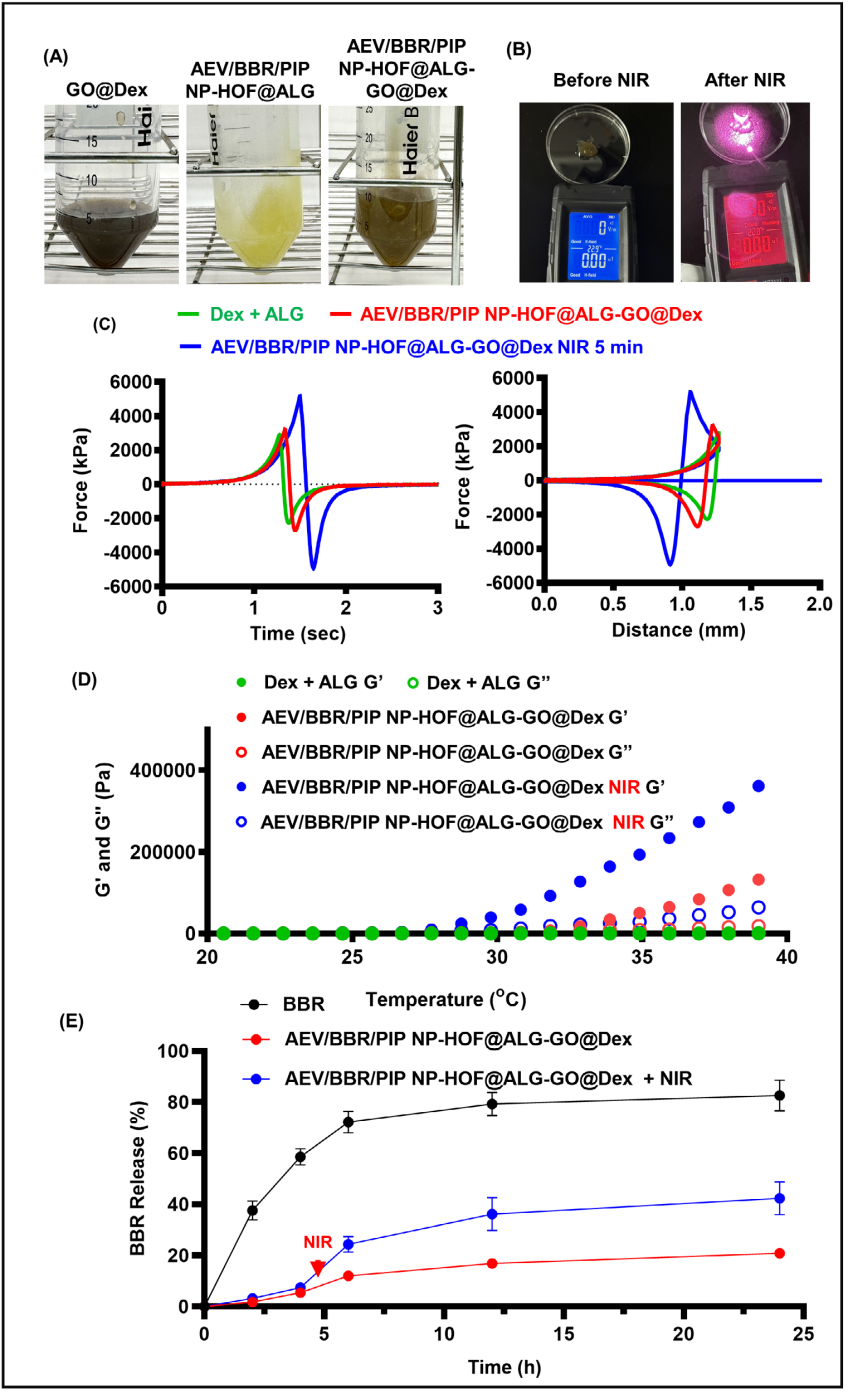
Material characterization and functional analysis of AEV/BBR/PIP NP‐HOF@ALG‐GO@Dex. (A) Photographs of prepared samples: GO@Dex, AEV/BBR/PIP NP‐HOF@ALG, and AEV/BBR/PIP NP‐HOF@ALG‐GO@Dex. (B) Electromagnetic responses of AEV/BBR/PIP NP‐HOF @ALG‐GO@Dex before and after NIR irradiation. (C) Texture analysis (TA). (D) Rheological properties of Dex+ALG and AEV/BBR/PIP NP‐HOF@ALG‐GO@Dex with and without NIR exposure, highlighting the structural stability under photothermal activation. (E) BBR release profiles of AEV/BBR/PIP NP‐HOF@ALG‐GO@Dex with and without NIR irradiation, showing controlled and accelerated release upon photothermal stimulation.

Figure 2A displays photographic evidence of the prepared materials, including GO@Dex, AEV/BBR/PIP NP‐HOF@ALG, and AEV/BBR/PIP NP‐HOF@ALG‐GO@Dex. Each sample exhibited distinct visual characteristics, with the HOF‐containing hydrogel formulation (AEV/BBR/PIP NP‐HOF@ALG‐GO@Dex) showing a homogeneous appearance, indicative of the successful integration of all components. Figure 2B illustrates the electromagnetic activities of AEV/BBR/PIP NP‐HOF@ALG‐GO@Dex before and after NIR irradiation. Upon NIR exposure, a notable increase in electromagnetic activity was observed.

The TA results in Figure 2C reveal mechanical differences between Dex+ALG and AEV/BBR/PIP NP‐HOF@ALG‐GO@Dex, with and without NIR irradiation for 5 min. Force–time and force–distance curves illustrate the impacts of material composition and external stimuli on the mechanical properties. The Dex+ALG formulation exhibited a rather weak force response, indicative of a relatively soft and elastic hydrogel structure. However, after incorporating AEV/BBR/PIP NP‐HOF@ALG‐GO@Dex, the force response increased, suggesting a more rigid and structured network. This change was likely due to the presence of GO and HOF‐based nanostructures, which may have enhanced intermolecular interactions and overall network cohesion. A particularly striking effect was observed when NIR irradiation was applied to AEV/BBR/PIP NP‐HOF@ALG‐GO@Dex, where the force magnitude further increased, demonstrating a clear NIR‐triggered enhancement in mechanical strength. This phenomenon suggests that NIR‐responsive heating may have promoted structural rearrangement within the polymeric network, possibly through localized enhancement of intermolecular interactions. The mechanical reinforcement observed after NIR exposure is consistent with stimuli‐responsive adaptability of the material and may be relevant to applications requiring tunable viscoelastic properties.

The rheological analysis compared the structural integrity of Dex+ALG and AEV/BBR/PIP NP‐HOF@ALG‐GO@Dex with and without NIR exposure. Before irradiation, both materials exhibited typical gel‐like behavior with stable shear‐thinning properties. However, upon NIR exposure, AEV/BBR/PIP NP‐HOF @ALG‐GO@Dex exhibited increases in viscosity and storage modulus, consistent with NIR‐triggered structural reinforcement of the matrix, potentially associated with photo‐activation of the GO‐containing network (Figure 2D). This enhanced mechanical stability under photothermal conditions supports the applicability of the formulation in dynamic biological environments. The BBR release profile (Figure 2E) was evaluated for AEV/BBR/PIP NP‐HOF@ALG‐GO@Dex under NIR irradiation. Without NIR exposure, a controlled, gradual release of BBR was observed, indicating the material's capacity for sustained delivery. Upon NIR irradiation, the cumulative BBR release was markedly higher than that of the non‐irradiated condition over the observation period. This enhanced release under photo‐activation was likely due to localized heating effects, which facilitated diffusion of the encapsulated drugs. In contrast, pure BBR showed a rapid release profile, lacking the controlled delivery properties offered by the hydrogel matrix.

Franz cell release analysis at the 30‐min time point showed a baseline fluorescence intensity for free BBR. Incorporation into AEV/BBR NP‐HOF@ALG‐GO@Dex produced a slight increase, whereas hydrogel containing AEV/BBR/PIP NP‐HOF@ALG‐GO@Dex displayed a notably higher release. The greatest release was observed for the AEV/BBR/PIP NP‐HOF@ALG‐GO@Dex under NIR irradiation, confirming that both PIP incorporation and NIR stimulation collectively enhanced transdermal BBR diffusion (Figure S2).

### 2.4 Integrated Photo‐Redox‐Responsive Properties of AEV/BBR/PIP NP‐HOF@ALG‐GO@Dex

**FIGURE 3:**
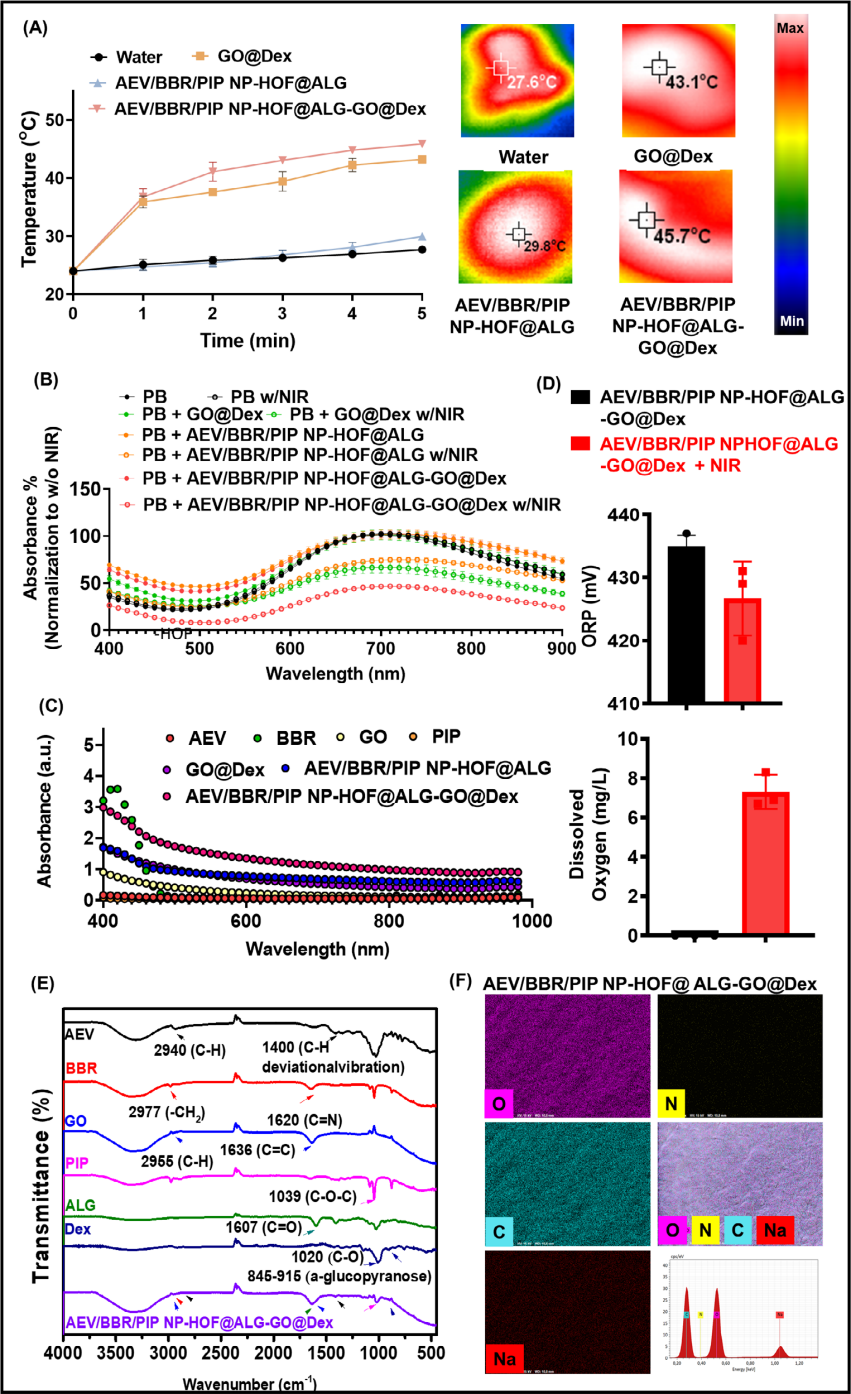
Photothermal, electrochromic, and redox properties of AEV/BBR/PIP NP‐HOF@ALG‐GO@Dex. (A) Temperature profiles of different samples under NIR irradiation, showing the photothermal response of the GO‐containing systems. (B) UV–vis absorbance changes of Prussian blue solutions, indicating light‐responsive redox‐related changes in the Fe^3^^+^/Fe^2^^+^ system. (C) The spectrometric data showed distinct UV–vis spectral features among the different groups. (D, above) ORP measurements and (D, below) dissolved oxygen profiles of AEV/BBR/PIP NP‐HOF@ALG‐GO@Dex with or without NIR irradiation, supporting NIR‐associated redox‐related and oxygen‐profile changes in the formulation. (E) FTIR spectra illustrating characteristic functional groups and their interactions within the composite matrix, supporting the integrated structure of the formulation. (F) SEM micrograph showing a porous network structure of the composite, together with EDS mapping indicating the distribution of key elements (C, O, N, Na), providing structural evidence for the integrated composite architecture.

Figure 3A demonstrates the photothermal behavior of AEV/BBR/PIP NP‐HOF@ALG‐GO@Dex under NIR irradiation compared to control samples. The GO@Dex‐based system exhibited a notable temperature increase, reaching approximately 43.2°C within 5 min of NIR exposure. In contrast, AEV/BBR/PIP NP‐HOF@ALG showed a moderate temperature rise (to ∼29.9°C), reflecting the absence of GO's photothermal properties [22]. The AEV/BBR/PIP NP‐HOF@ALG‐GO@Dex hybrid, however, reached the highest temperature (∼45.9°C), consistent with enhanced photothermal responsiveness in the formulation containing both GO and HOF‐related components [23]. This temperature range is consistent with mild hyperthermia, which may contribute to barrier modulation and transport enhancement and may be relevant to the downstream biological responses observed in this study, without indicating thermal ablation. The reproducible temperature increase observed in this system suggests its suitability for controlled photothermal intervention under the tested conditions.

In the context of this study, NIR‐triggered heating is interpreted as mild hyperthermia rather than ablative photothermal therapy, and its primary relevance is discussed in terms of barrier modulation, transport facilitation, and microenvironment‐associated responses. Accordingly, the therapeutic relevance of this temperature range in the present study is interpreted primarily in relation to surface‐associated heating, transient barrier loosening, and improved transdermal transport, rather than direct bulk heating of deep joint tissues.

The UV–vis spectroscopic analysis (Figure 3B) revealed significant changes in the absorbance of electrochromic Prussian blue (PB) solutions (Fe^3^^+^ to Fe^2^^+^ transitions) upon NIR irradiation. In the GO@Dex system, NIR‐triggered photochemical effects reduced Fe^3^^+^ to Fe^2^^+^, as indicated by a decrease in the absorbance at about 700 nm. The AEV/BBR/PIP NP‐HOF@ALG‐GO@Dex system exhibited the most pronounced absorbance change, suggesting a stronger light‐responsive electrochemical response in this formulation. This reduction is consistent with light‐responsive electrochemical behavior in the GO‐containing system and suggests the possibility of altered interfacial electron‐transfer–related processes under the tested conditions. The observed electrochromic changes suggest light‐responsive electrochemical activity in the system, which may contribute to the modulation of inflammatory responses in complex biological environments. The spectrometric data (Figure 3C) revealed distinct UV–vis absorption characteristics among the different materials.

Figure 3D (above) evaluates the oxidative‐reductive environment of AEV/BBR/PIP NP‐HOF@ALG‐GO@Dex with and without NIR irradiation. Without irradiation, the oxygen reduction potential (ORP) remained stable at approximately 435 mV, indicative of a neutral redox environment. Upon NIR exposure, the ORP decreased to 400 mV, reflecting enhanced reductive activity likely mediated by GO's photoelectrochemical properties. Such a redox‐associated microenvironment may contribute to ROS attenuation and thereby support the mitigation of oxidative stress under inflammatory conditions. The dissolved oxygen data (below, Figure 3D) further suggested that NIR irradiation altered the oxygen‐related behavior of the system. Under NIR, AEV/BBR/PIP NP@ALG‐GO@Dex showed a quantifiable increase in dissolved oxygen relative to the non‐irradiated condition. The quantified increase in dissolved oxygen under NIR supports oxygen‐related physicochemical modulation of the formulation under the tested conditions. Nevertheless, this result should be interpreted cautiously, as it does not by itself establish that the observed magnitude of oxygen increase is biologically sufficient to relieve hypoxia in deep joint tissue in vivo.

Under NIR, AEV/BBR/PIP NP@ALG‐GO@Dex produced a marked increase in dissolved oxygen concentrations. Consistent with prior reports describing light‐responsive oxygen‐related changes in GO‐containing systems [24], an increase in dissolved oxygen was observed under the present experimental conditions.

This observation suggests that the GO‐containing system may be associated with NIR‐responsive changes in the local hypoxia‐ and redox‐related microenvironment, which could provide a supportive physicochemical context for the observed biological effects. Together with the photothermal and electrochemical responsiveness of the formulation, these findings support the multifunctional character of the platform while not by themselves establishing direct causal molecular pathways for the downstream biological effects.

Importantly, these physicochemical observations do not by themselves establish direct tissue oxygenation in vivo or define whether the observed oxygen increase is biologically sufficient to relieve hypoxia in deep joint tissues.

### 2.5 Molecular Interactions and Elemental Distribution in AEV/BBR/PIP NP‐HOF@ALG‐GO@Dex

Fourier transform IR (FTIR) spectra (Figure 3E) provide detailed insights into the molecular interactions within the composite. The individual spectra of AEV, BBR, PIP, GO, Dex, and ALG all displayed characteristic functional group peaks. Specifically, BBR exhibited characteristic bands near 2977 and 1620 cm^−^^1^, while AEV showed representative signals at ∼2940 and 1400 cm^−^^1^.The PIP showed the peaks 1039 cm^−1^.  GO displayed characteristic features around 2966 and 1620 cm^−^^1^, whereas ALG showed a prominent band at 1607 cm^−^^1^. Dex was characterized by a peak at ∼1020 cm^−^^1^ together with α‐glucopyranose‐associated signals in the 845–915 cm^−^^1^ region, consistent with the expected spectral features of these components. In the AEV/BBR/PIP NP‐HOF@ALG‐GO@Dex composite, several overlapping peaks with slight shifts were observed, confirming the successful incorporation of these components into a single, stable matrix. The broad O–H stretching region further indicates the hydrophilic character of the composite, which may be favorable for formulation stability and subsequent delivery‐related applications. Figure 3F presents morphological and elemental compositions of the AEV/BBR/PIP NP‐HOF@ALG‐GO@Dex composite as analyzed by SEM coupled with EDS. The SEM micrograph reveals a uniform, porous network structure, ideal for drug encapsulation and sustained release. This porous architecture ensures a high surface area and facilitates efficient interaction with the biological microenvironment.

EDS mapping confirmed the presence of key elements, including carbon (C), oxygen (O), nitrogen (N), and sodium (Na), consistent with the incorporation of Dex, ALG, GO, and AEVs. The relatively uniform elemental distribution within the matrix supports homogeneous integration of the individual components. Combined with the SEM observations, these results provide structural evidence for the integrated composite architecture and its suitability as a multifunctional formulation platform for further biological assessment.

The NMR spectra in Figure S1D provides a comprehensive molecular characterization of AEV/BBR/PIP NP‐HOF@ALG‐GO@Dex. The AEV/BBR/PIP NP‐HOF@ALG‐GO@Dex composite exhibited characteristic proton signals corresponding to its individual components. BBR contributed aromatic proton signals in the 7.0–9.8 ppm range, associated with its isoquinoline alkaloid framework. PIP showed notable peaks in the 1.2–2.2 ppm aliphatic region, corresponding to the piperidine ring protons. ALG displayed broad peaks centered at 3.7–50. ppm, with anomeric proton signals near 8.4 ppm, reflecting its polysaccharide backbone. Dex contributed distinct resonances in the 3.4–5.3 ppm hydroxyl‐rich region, consistent with its complex polysaccharide structure. GO showed characteristic signals in the 2.3‐4.7 ppm region, indicative of hydroxyl and epoxide functional groups. In the composite spectrum, the polysaccharide resonances of ALG and Dex were notably broadened in the 3–6 ppm range, suggesting strong molecular entanglement within the gel formulation.

### 2.6 In Vitro Cytotoxicity and Dose‐Dependent Cellular Tolerance of AEV/BBR/PIP NP‐HOF@ALG‐GO@Dex

**FIGURE 4:**
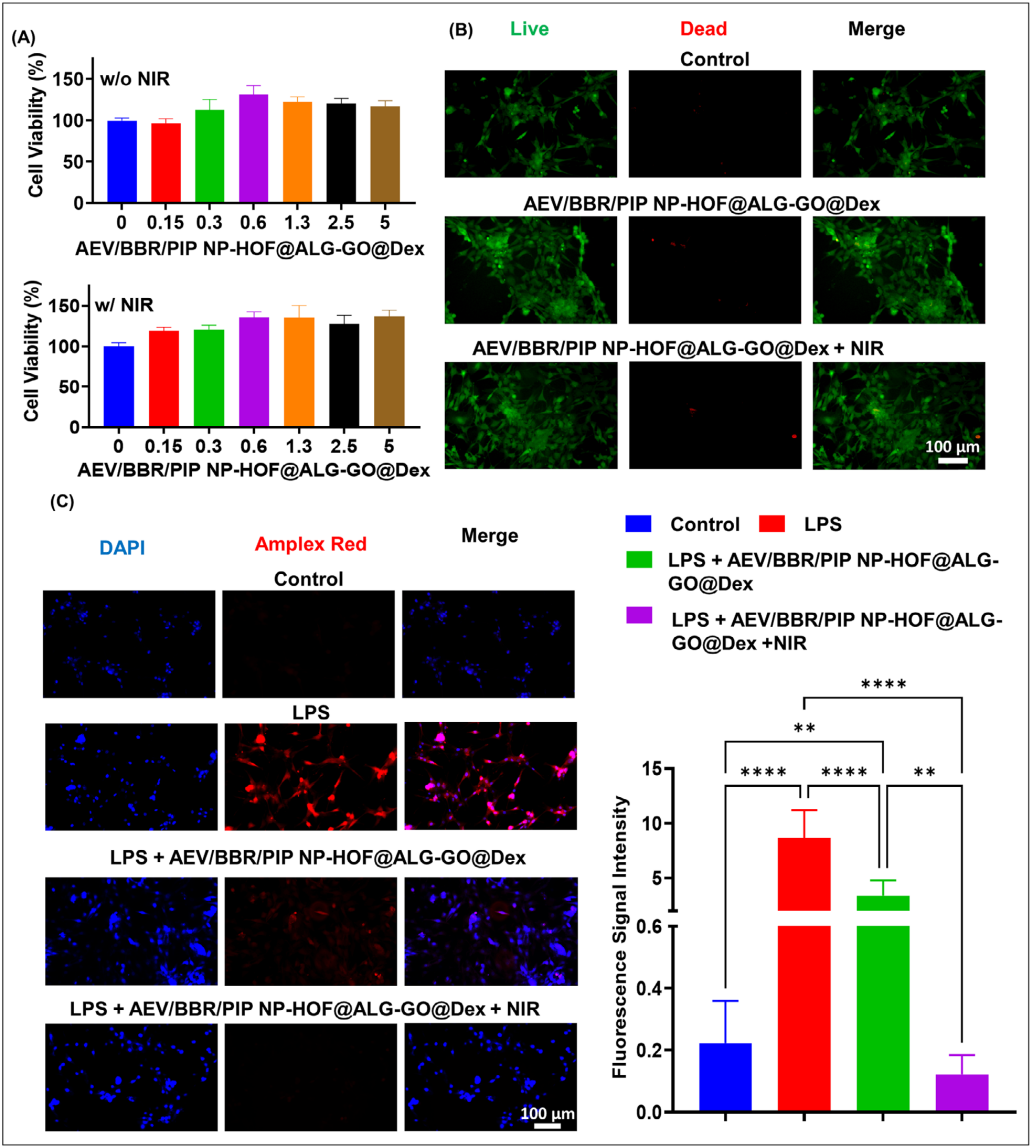
Biocompatibility evaluation of AEV/BBR/PIP NP‐HOF@ALG‐GO@Dex. (A) Cell viability as assessed by an MTT assay at various concentrations (0, 0.15, 0.3, 0.6, 1.3, 2.5, and 5 mg/mL) with and without NIR irradiation. (B) Fluorescence microscopic images of live/dead‐stained cells treated with nanoparticles at different concentrations, with and without NIR irradiation. Green indicates live cells, and red indicates dead cells. (C) Amplex red fluorescence images and quantitative analysis showing ROS levels under different treatment conditions, with and without NIR irradiation. Statistical significance was defined as *p* < 0.05 (^*^), *p* < 0.01 (^**^), *p* < 0.001 (^***^), and *p* < 0.0001 (^****^).

The biocompatibility of AEV/BBR/PIP NP‐HOF@ALG‐GO@Dex at various concentrations (0, 0.15, 0.3, 0.6, 1.3, 2.5, and 5 mg/mL) toward chondrocytes was assessed through MTT assays (Figure 4A) and live/dead fluorescence microscopic imaging (Figure 4B), both with and without NIR irradiation. These investigations were aimed at elucidating the cytotoxicity profile and therapeutic potential of this composite system. MTT assays indicated a concentration‐dependent effect of AEV/BBR/PIP NP‐HOF@ALG‐GO@Dex on cell viability.

Interestingly, under NIR irradiation, cell viability remained above 100% even at the highest tested concentration of 5 mg/mL, supporting the overall biocompatibility of the composite system under the applied experimental conditions. This finding may reflect treatment‐associated metabolic activation or adaptive cellular responses under mild hyperthermia conditions, rather than indicating a direct proliferative conclusion. Accordingly, the MTT result is interpreted here as evidence of preserved metabolic viability rather than as proof of enhanced cell growth.

### 2.7 Antioxidant Effects of AEV/BBR/PIP NP‐HOF@ALG‐GO@Dex‐ in an In Vitro Chondrocyte Inflammation and Immune Cells Hypoxia Model

The effects of AEV/BBR/PIP NP‐HOF@ALG‐GO@Dex on oxidative stress in LPS‐stimulated chondrocyte cells were assessed using Amplex red fluorescence imaging (Figure 4C). Without NIR irradiation, treatment with AEV/BBR/PIP NP‐HOF@ALG‐GO@Dex reduced ROS levels compared to that of the LPS‐only controls, as indicated by a marked decrease in the red fluorescence intensity. This reduction is consistent with the antioxidant‐related activity of AEV, BBR, and PIP. Following NIR irradiation, ROS levels were further decreased, suggesting an association between NIR‐responsive behavior in the GO‐containing formulation and additional attenuation of oxidative stress under the tested conditions. Quantitative analysis (Figure 4C) revealed lower ROS levels in the AEV/BBR/PIP NP‐HOF@ALG‐GO@Dex+NIR group than in both the LPS‐only and non‐irradiated AEV/BBR/PIP NP‐HOF@ALG‐GO@Dex groups, suggesting that NIR activation may further enhance oxidative stress attenuation in the formulation. These findings provide supportive indication for the immune‐related modulatory potential of AEV/BBR/PIP NP‐HOF@ALG‐GO@Dex. The enhanced antioxidant‐ and inflammation‐related effects observed under NIR stimulation suggest that light‐responsive redox‐associated changes in the nanocomposite may be associated with improved regulation of ROS‐related inflammatory responses, although direct pathway‐level links remain to be clarified.

To further evaluate the biological relevance of the GO‐containing system under NIR stimulation in a controlled disease‐relevant context, an additional hypoxia‐based RAW macrophage experiment was performed (Figure S3). Compared with hypoxia alone and hypoxia + GO conditions, the hypoxia + GO + NIR group showed a more pronounced shift toward M2‐associated polarization, together with reduced ROS levels and attenuated HIF‐associated signaling. These findings strengthen the interpretation that NIR‐responsive activity in the GO‐containing system is biologically associated with modulation of inflammatory and hypoxia‐related responses under hypoxic conditions. Nevertheless, these data are interpreted as supportive mechanistic evidence rather than as definitive proof of a fully resolved downstream molecular pathway.

### 2.8 Immunomodulatory Effects of AEV/BBR/PIP NP‐HOF@ALG‐GO@Dex on Macrophage Polarization

**FIGURE 5:**
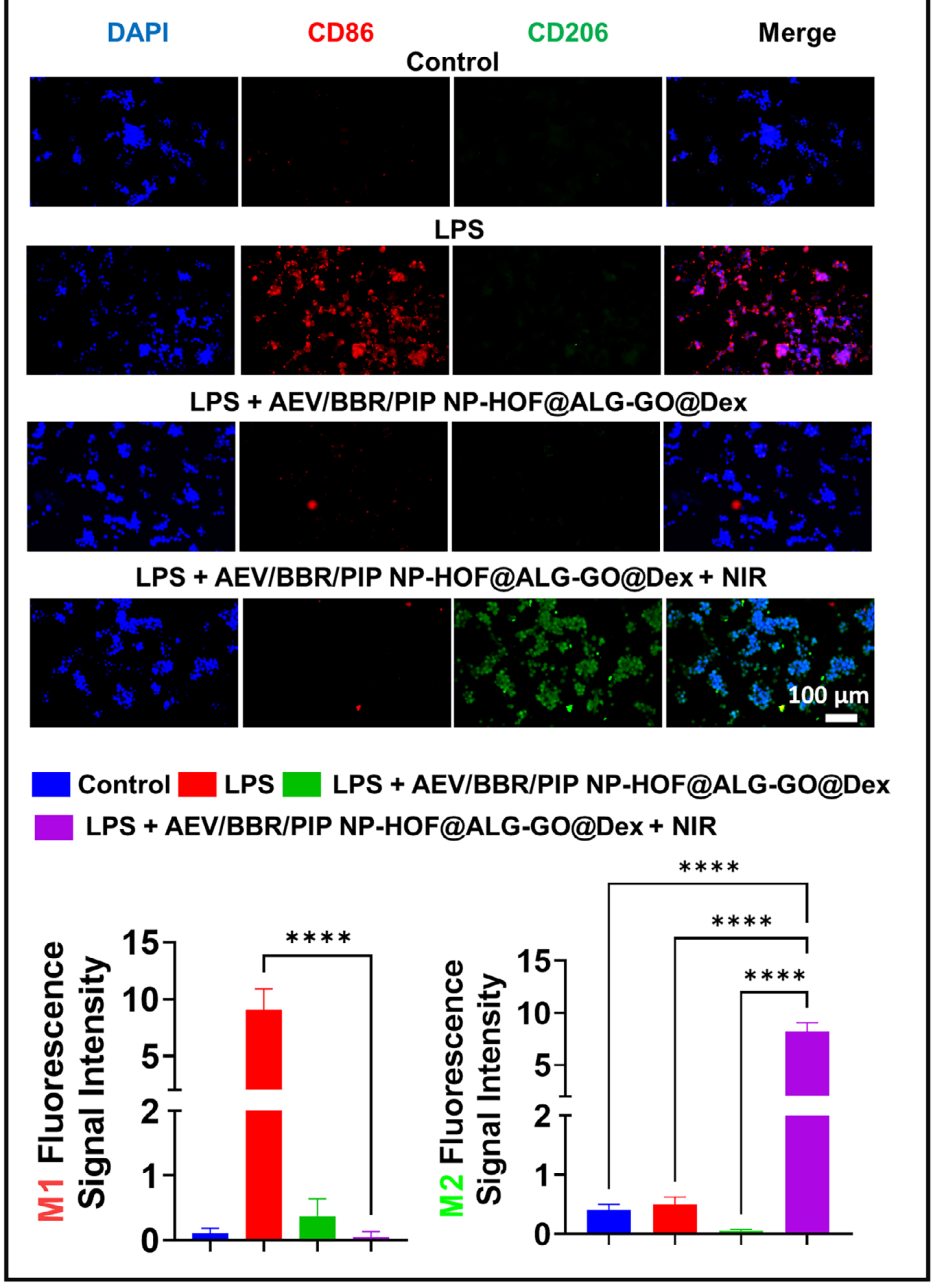
Macrophage polarization analysis of LPS‐stimulated RAW cells treated with AEV/BBR/PIP NP‐HOF@ALG‐GO@Dex. IF images showing DAPI (blue), CD86 (red, M1 marker), and CD206 (green, M2 marker) under different treatment conditions. Quantitative analysis of CD86 and CD206 expression across treatment groups (LPS, LPS+AEV/BBR/PIP NP‐HOF@ALG‐GO@Dex, and LPS+AEV/BBR/PIP NP‐HOF@ALG‐GO@Dex+NIR), indicating a more pronounced shift toward M2‐associated polarization under NIR irradiation. Scale bars: 100 µm. Statistical significance was defined as *p* < 0.05 (^*^), *p* < 0.01 (^**^), *p* < 0.001 (^***^), and *p* < 0.0001 (^****^).

Macrophage polarization in RAW cells was assessed by IF imaging through analysis of markers associated with the proinflammatory M1 and anti‐inflammatory M2 phenotypes (Figure 5, above), specifically analyzing expressions of the M1 marker, CD86 (red), and M2 marker, CD206 (green) [21]. The experimental groups included LPS alone, LPS+AEV/BBR/PIP NP‐HOF@ALG‐GO@Dex, and LPS+AEV/BBR/PIP NP‐HOF @ALG‐GO@Dex+NIR irradiation.

In the LPS‐treated group, high expression of CD86 was observed, accompanied by minimal CD206 expression, indicative of dominant M1 polarization. Treatment with AEV/BBR/PIP NP‐HOF@ALG‐GO@Dex reduced CD86 expression and modestly increased CD206 levels, suggesting a partial shift toward M2 polarization. Upon NIR irradiation (in the AEV/BBR/PIP NP‐HOF@ALG‐GO@Dex+NIR group), a significant increase in CD206 fluorescence and a pronounced reduction in CD86 expression were observed, indicating that NIR‐responsive activity in the GO‐containing system was associated with a more pronounced shift toward M2‐associated polarization.

Quantification of the fluorescence intensity (Figure 5, below) revealed a reduction in CD86 expression in the LPS+AEV/BBR/PIP NP‐HOF@ALG‐GO@Dex group compared to the LPS‐only group, while NIR irradiation (in the AEV/BBR/PIP NP‐HOF@ALG‐GO@Dex+NIR group) further suppressed CD86 levels. Concurrently, CD206 expression increased by approximately 8.25‐fold in the LPS+AEV/BBR/PIP NP‐HOF@ALG‐GO@Dex+NIR group compared to the other groups. These results indicate that NIR irradiation was associated with an enhanced immunomodulatory profile of the nanocomposite, as reflected by a more pronounced shift in macrophage polarization. The observed effects are likely associated with the combined contributions of multiple functional components within the formulation. Among these, Piperine components may have contributed, at least in part, to the anti‐inflammatory and antioxidant‐related activity [25], while BBR suppressed angiogenesis [26] and PIP enhanced macrophage phenotype modulation. The GO‐containing system may further contribute to the observed M2‐associated polarization under NIR stimulation by modulating the local microenvironment in a manner favorable to anti‐inflammatory responses. Additionally, the altered oxygen‐related behavior observed in the GO‐containing system may be relevant to the modulation of hypoxia‐associated responses, although direct causal linkage was not established in the present study [27]. These findings support the potential of AEV/BBR/PIP NP‐HOF@ALG‐GO@Dex as a platform for immune‐related modulation. The ability to fine‐tune macrophage polarization under NIR irradiation suggests its potential application in treating chronic inflammatory conditions, such as rheumatoid arthritis, cystitis, and atherosclerosis. Future studies could explore the in vivo implications of this system and its long‐term effects on systemic immunity.

### 2.9 Transdermal Therapeutic Performance of AEV/BBR/PIP NP‐HOF@ALG‐GO@Dex in an In Vivo OA Rat Model

**FIGURE 6:**
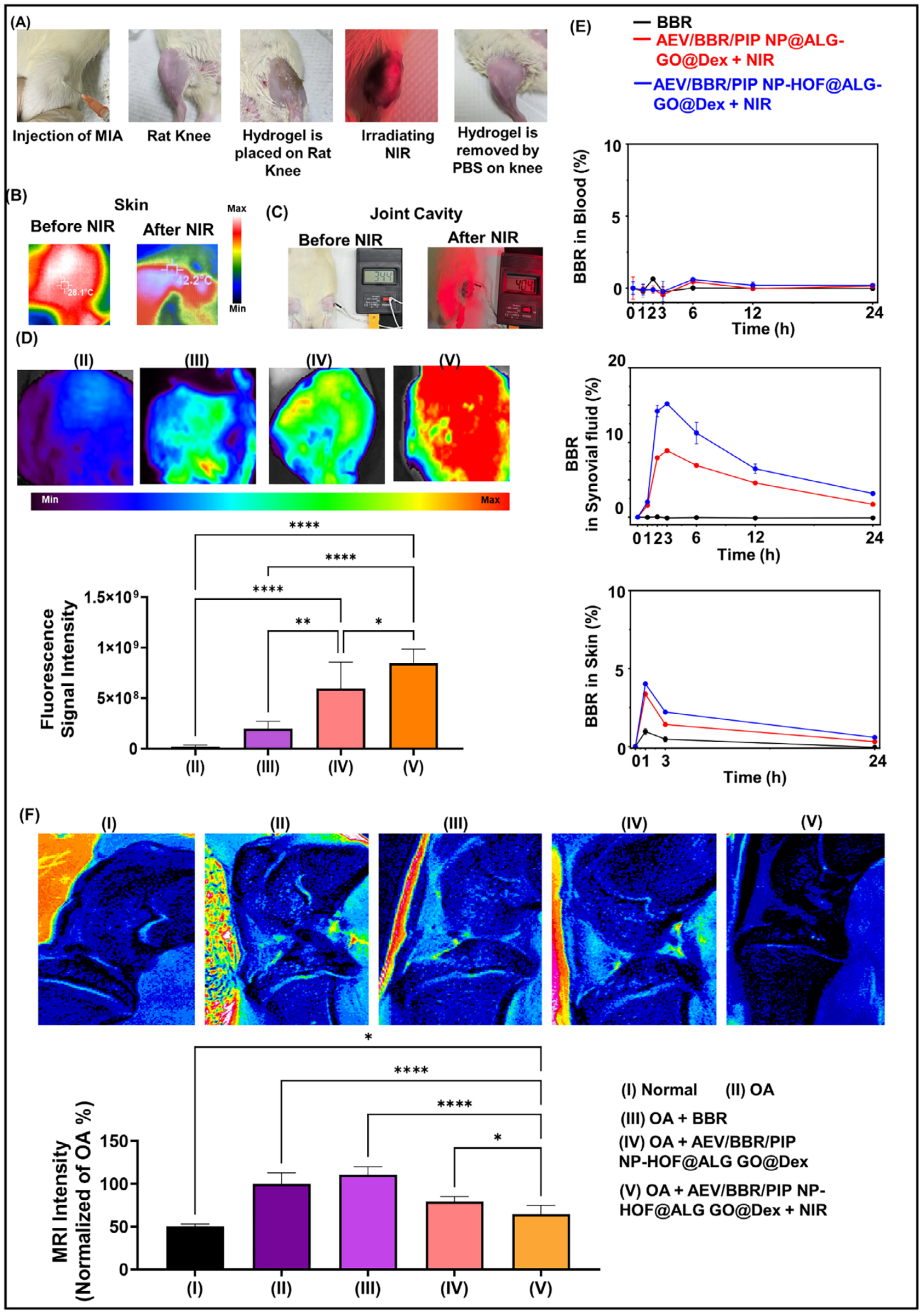
In vivo evaluation of transdermally delivered AEV/BBR/PIP NP‐HOF@ALG‐GO@Dex in a rat model of MIA‐induced osteoarthritis. (A) Photographic representation of the MIA‐induced knee OA model, transdermal administration, and NIR irradiation. (B) Infrared thermal imaging showing localized surface heating at the knee joint following NIR irradiation. (C) Photographic verification of thermocouple placement in the cartilage region and corresponding temperature readout, showing deep‐region thermal responses under the same treatment and irradiation conditions. (D) IVIS fluorescence imaging and quantification of BBR retention after hydrogel removal, demonstrating enhanced transdermal delivery in the nanoparticle‐ and NIR‐treated groups. (E) Time‐dependent quantification of BBR levels in blood, skin, and joint‐associated tissue after treatment, showing minimal systemic exposure and improved local tissue retention, particularly in the HOF‐containing formulation. (F) T2‐weighted MRI (color‐scale, high to low: red, yellow, green, and blue) and normalized grayscale analysis of knee joints, showing attenuation of edema‐ and inflammation‐associated signals following treatment, with the greatest reduction observed in the HOF‐containing formulation combined with NIR irradiation. Statistical significance was defined as *p* < 0.05 (^*^), *p* < 0.01 (^**^), *p* < 0.001 (^***^), and *p* < 0.0001 (^****^).

As shown in the photographic data (Figure 6A), a rat model of MIA‐induced osteoarthritis was employed to evaluate the therapeutic effects of transdermally delivered AEV/BBR/PIP NP‐HOF@ALG‐GO@Dex with or without NIR stimulation. Thermal imaging (Figure 6B) showed a clear local temperature increase at the knee joint following NIR irradiation, with the AEV/BBR/PIP NP‐HOF@ALG‐GO@Dex + NIR group reaching approximately 42.2°C, whereas the corresponding non‐irradiated group remained at 28.1°C. This rise in temperature confirms the efficient photothermal conversion of the GO‐containing nanocomposite. Importantly, the observed temperature remains within the widely recognized mild‐hyperthermia therapeutic range, which is known to suppress inflammatory signaling while avoiding thermal injury, thereby supporting the potential of NIR‐assisted modulation of the OA microenvironment.

Because the measured thermal signal reflects surface temperature at the knee region, these data should not be interpreted as direct evidence of equivalent temperature elevation within deep cartilage or synovial tissue. Rather, the therapeutic relevance of NIR treatment in this study is discussed primarily in relation to surface‐mediated barrier modulation and improved transdermal delivery.

To address this point more directly, we additionally performed temperature monitoring using a thermocouple positioned within the cartilage region under the same irradiation conditions used in the therapeutic study. These measurements enabled comparison between surface heating and deeper tissue temperature elevation. The photographic data confirm thermocouple placement in the cartilage of treated animals (AEV/BBR/PIP NP‐HOF@ALG‐GO@Dex + NIR, Figure 6C), while the corresponding thermal readout indicated that the cartilage‐region temperature reached approximately 40°C. This temperature was lower than the surface temperature measured by thermal imaging, further supporting a cautious interpretation in which the therapeutic effects are attributed primarily to surface‐associated heating, transient barrier loosening, and enhanced transdermal delivery, with possible but limited contribution from deeper thermal effects.

Importantly, ‘effective temperature’ should not be interpreted as a strict binary threshold that must be maintained for a prolonged period. Transdermal transport is governed by temperature‐dependent diffusivity/partitioning and barrier resistance; even transient mild hyperthermia within the 40°C–45°C range can measurably increase molecular mobility and transiently reduce stratum corneum barrier resistance, thereby enhancing net permeation beyond what would be inferred from endpoint temperature alone.

Skin/joint‐surface temperature (Figure S4) during irradiation was recorded continuously to generate a full temperature–time profile (in addition to endpoint thermograms), and the measurement position was fixed at the center of the irradiated knee region for all animals to ensure comparability.

IVIS fluorescence imaging (Figure 6D) was subsequently conducted to assess the distribution of BBR following transdermal administration. To avoid any optical interference from residual material on the skin surface, the hydrogel layer was washed/removed prior to imaging, ensuring accurate quantification of fluorescence originating from retention. In the BBR‐only group, fluorescence signals were relatively weak and diffuse, suggesting poor penetration and retention. In contrast, AEV/BBR/PIP NP‐HOF@ALG‐GO@Dex slightly enhanced the fluorescence intensity, indicating improved drug delivery and accumulation around the joint. AEV/BBR/PIP NP‐HOF@ALG‐GO@Dex+NIR irradiation further amplified the fluorescence signal, demonstrating the combined effect of the photothermal response in enhancing transdermal drug penetration.

To more directly quantify local delivery beyond fluorescence‐based IVIS imaging, BBR levels were additionally measured in blood, skin, and joint‐associated tissue after treatment (Figure 6E). Blood BBR levels remained minimal across the examined time points, indicating low systemic exposure. In contrast, measurable BBR accumulation was observed in joint‐associated tissue, with peak levels at 2–3 h after administration followed by a gradual decline. Importantly, the HOF‐containing hydrogel (AEV/BBR/PIP NP‐HOF@ALG‐GO@Dex) + NIR group showed higher joint‐associated BBR levels than the non‐HOF hydrogel (AEV/BBR/PIP NP@ALG‐GO@Dex) + NIR group, while free BBR exhibited the lowest tissue retention. In skin tissue, BBR levels peaked at 1 h and declined thereafter, consistent with an initial local depot followed by redistribution and clearance. Together, these findings provide direct concentration‐based support for local BBR delivery to the joint‐associated region and for improved retention in the HOF‐containing formulation.

MRI (Figure 6F) was used to assess the severity of joint edema and inflammation in the knee. T2‐weighted images showed bright white to color‐mapped signals, indicative of increased water retention and inflammatory changes in MIA‐induced OA joints. Notably, the OA and OA+BBR groups exhibited strong signal intensities, suggesting pronounced edema and severe inflammatory involvement. Treatment with AEV/BBR/PIP NP‐HOF@ALG‐GO@Dex slightly reduced these signals, with NIR irradiation further suppressing inflammation. Quantitative analysis of normalized MRI grayscale intensity revealed an approximately 20% decrease in edema‐associated signal in the AEV/BBR/PIP NP‐HOF@ALG‐GO@Dex group and an approximately 35% decrease in the AEV/BBR/PIP NP‐HOF@ALG‐GO@Dex + NIR group compared with the OA group. These results indicate that the nanocomposite system, particularly when combined with NIR activation, may contribute to attenuation of inflammatory features and edema in the treated joints.

The observed therapeutic outcomes are likely associated with the combined functional contributions of the components within AEV/BBR/PIP NP‐HOF@ALG‐GO@Dex. AEVs may contribute antioxidant‐ and anti‐inflammatory–associated activity, whereas BBR and PIP may be involved in the observed modulation of macrophage polarization toward an anti‐inflammatory phenotype. In addition, the GO‐containing system may support NIR‐responsive local effects that are associated with improved transdermal transport and modulation of inflammatory responses. The Dex‐ALG hydrogel matrix ensured sustained release and retention of the therapeutic agents within the joint, enhancing the efficacy. Collectively, these findings support the potential of AEV/BBR/PIP NP‐HOF@ALG‐GO@Dex as a noninvasive, NIR‐responsive therapeutic platform for osteoarthritis management. The ability to combine localized photothermal therapy with targeted drug delivery offers a novel approach to managing joint inflammation and edema. Future studies should focus on long‐term in vivo evaluations and optimizing dosage regimens to ensure maximum therapeutic benefits while minimizing potential side effects.

### 2.10 Functional and Cognitive Effects of AEV/BBR/PIP NP‐HOF@ALG‐GO@Dex

**FIGURE 7:**
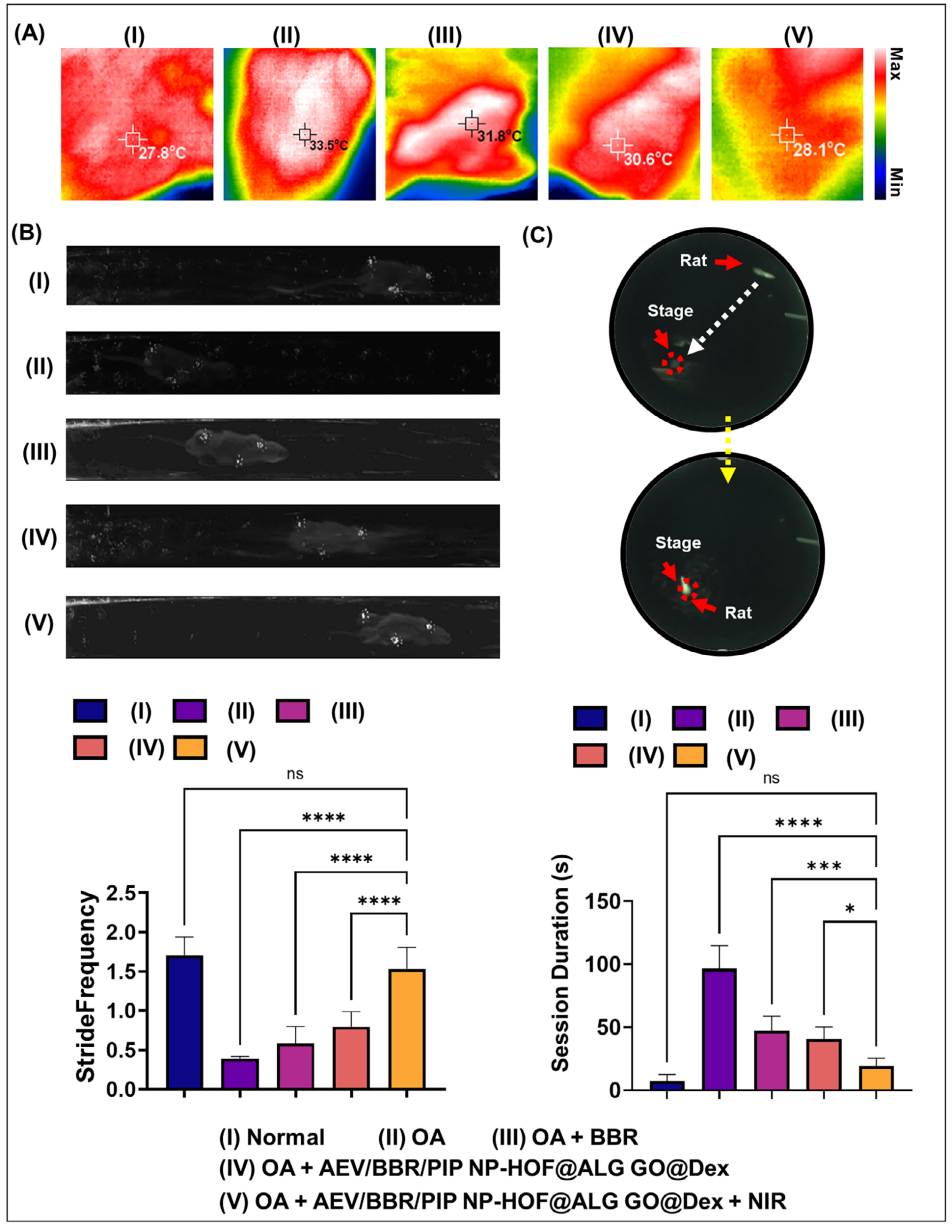
Evaluation of the therapeutic effects of AEV/BBR/PIP NP‐HOF@ALG‐GO@Dex in OA rats. (A) Thermal imaging of knee joint temperatures across different treatment groups (OA+BBR, OA+AEV/BBR/PIP NP‐HOF@ALG‐GO@Dex with/without NIR irradiation). (B) Gait analysis showing improvements in the frequency across groups, indicating functional recovery. (C) Morris water maze analysis demonstrating escape latency and platform‐targeting accuracy, evaluating the systemic effects of treatments on cognitive behavior. Statistical significance was defined as *p* < 0.05 (^*^), *p* < 0.01 (^**^), *p* < 0.001 (^***^), and *p* < 0.0001 (^****^).

The effects of transdermally administered AEV/BBR/PIP NP‐HOF@ALG‐GO@Dex, with and without NIR irradiation, on knee inflammation in OA rats were evaluated through long‐term skin temperature monitoring. In the OA+BBR group, the knee temperature remained elevated, indicating persistent inflammation (Figure 7A). In contrast, OA rats treated with AEV/BBR/PIP NP‐HOF@ALG‐GO@Dex showed a gradual reduction in temperature, with a more‐pronounced effect observed in the NIR‐treated group. By the end of the experimental period, knee temperatures in the NIR (AEV/BBR/PIP NP‐HOF@ALG‐GO@Dex) group were reduced to near‐normal levels (approximately 27.8°C), compared to 33.5°C in the non‐NIR group and 31.8°C in the BBR‐only group. These results suggest that the AEV/BBR/PIP NP‐HOF@ALG‐GO@Dex platform, particularly under NIR activation, may support attenuation of inflammatory responses through the combined contributions of the GO‐containing system and the bioactive components AEV, BBR, and PIP.

The therapeutic impact of the treatments on joint functionality was assessed using a gait analysis (Figure 7B). OA rats and OA rats in the BBR‐only group displayed relatively low gait frequencies, indicative of joint pain and limited mobility. Treatment with AEV/BBR/PIP NP‐HOF@ALG‐GO@Dex improved the gait frequency, with a marked improvement observed in the AEV/BBR/PIP NP‐HOF@ALG‐GO@Dex+NIR‐treated group. A quantitative analysis showed a significant increase in stride frequency (ratio) in the AEV/BBR/PIP NP‐HOF@ALG‐GO@Dex+NIR group compared to both the non‐NIR and BBR‐only groups. These findings suggest that treatment with AEV/BBR/PIP NP‐HOF@ALG‐GO@Dex under NIR irradiation was associated with improved joint function, potentially in relation to reduced inflammation and improved local tissue conditions.

To further evaluate behavioral performance in the OA model, rats were subjected to the Morris water maze test (Figure 7C). The OA+BBR group exhibited prolonged escape latency, whereas the AEV/BBR/PIP NP‐HOF@ALG‐GO@Dex‐treated groups showed improved performance in these parameters. The most pronounced improvement was observed in the additional NIR‐treated group. These results indicate an association between treatment and improved behavioral performance, but the mechanistic basis of this effect remains to be further investigated.

### 2.11 Immunomodulatory and Antioxidant Effects of AEV/BBR/PIP NP‐HOF@ALG‐GO@Dex With NIR Irradiation

**FIGURE 8:**
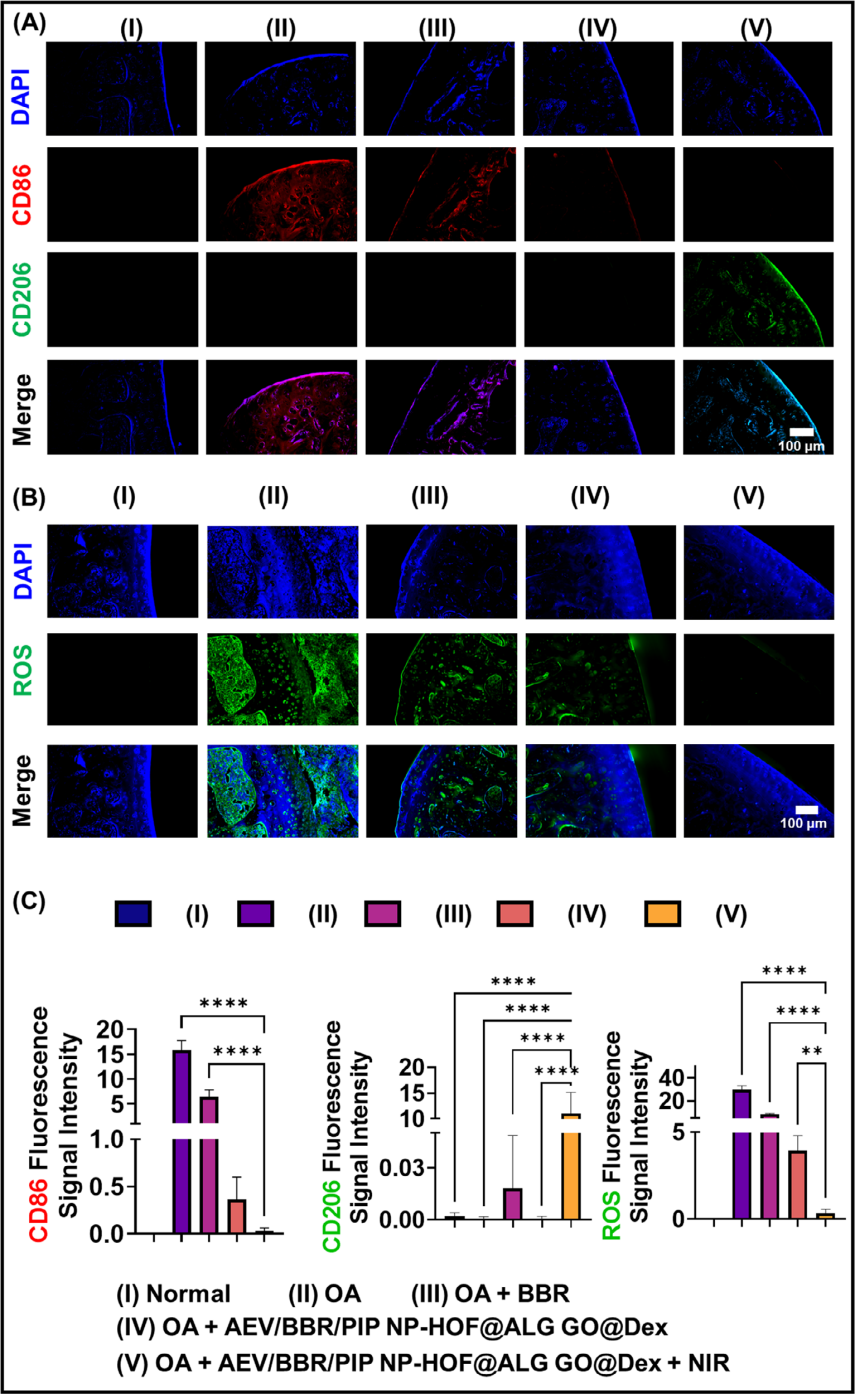
Immunomodulation and oxidative stress reduction by AEV/BBR/PIP NP‐HOF@ALG‐GO@Dex with NIR irradiation in OA cartilage. (A) IF staining for CD86 (an M1 marker, red) and CD206 (an M2 marker, green) showing significant M1‐to‐M2 macrophage polarization in the AEV/BBR/PIP NP‐HOF@ALG‐GO@Dex+NIR group compared to the other groups. (B) Reactive oxygen species (ROS) levels were assessed using the fluorescence intensity, revealing significant reductions in the NIR‐treated group. (C) Quantitative analysis. Statistical significance was defined as *p <* 0.05 (^*^), *p <* 0.01 (^**^), *p <* 0.001 (^***^), and *p <* 0.0001 (^****^). Scale bars: 100 µm.

The therapeutic activity of AEV/BBR/PIP NP‐HOF@ALG‐GO@Dex in the presence of NIR irradiation was further supported by immunofluorescence and oxidative stress analyses (Figure 8). IF staining suggested a shift in macrophage polarization within OA treated AEV/BBR/PIP NP‐HOF@ALG‐GO@Dex + NIR cartilage, as evidenced by increased CD206 expression and decreased CD86 levels (Figure 8A–C). These findings are consistent with modulation of the inflammatory microenvironment toward a more anti‐inflammatory state. Moreover, oxidative stress analysis revealed lower ROS levels (Figure 8B,C) together with a higher M2/M1 ratio in the AEV/BBR/PIP NP‐HOF@ALG‐GO@Dex+NIR‐treated group than in the OA, OA+BBR, and OA+AEV/BBR/PIP NP‐HOF@ALG‐GO@Dex groups.

### 2.12 Histological Assessment‐Microenvironmental Modulation Via AEV/BBR/PIP NP‐HOF@ALG‐GO@Dex Treatment

**FIGURE 9:**
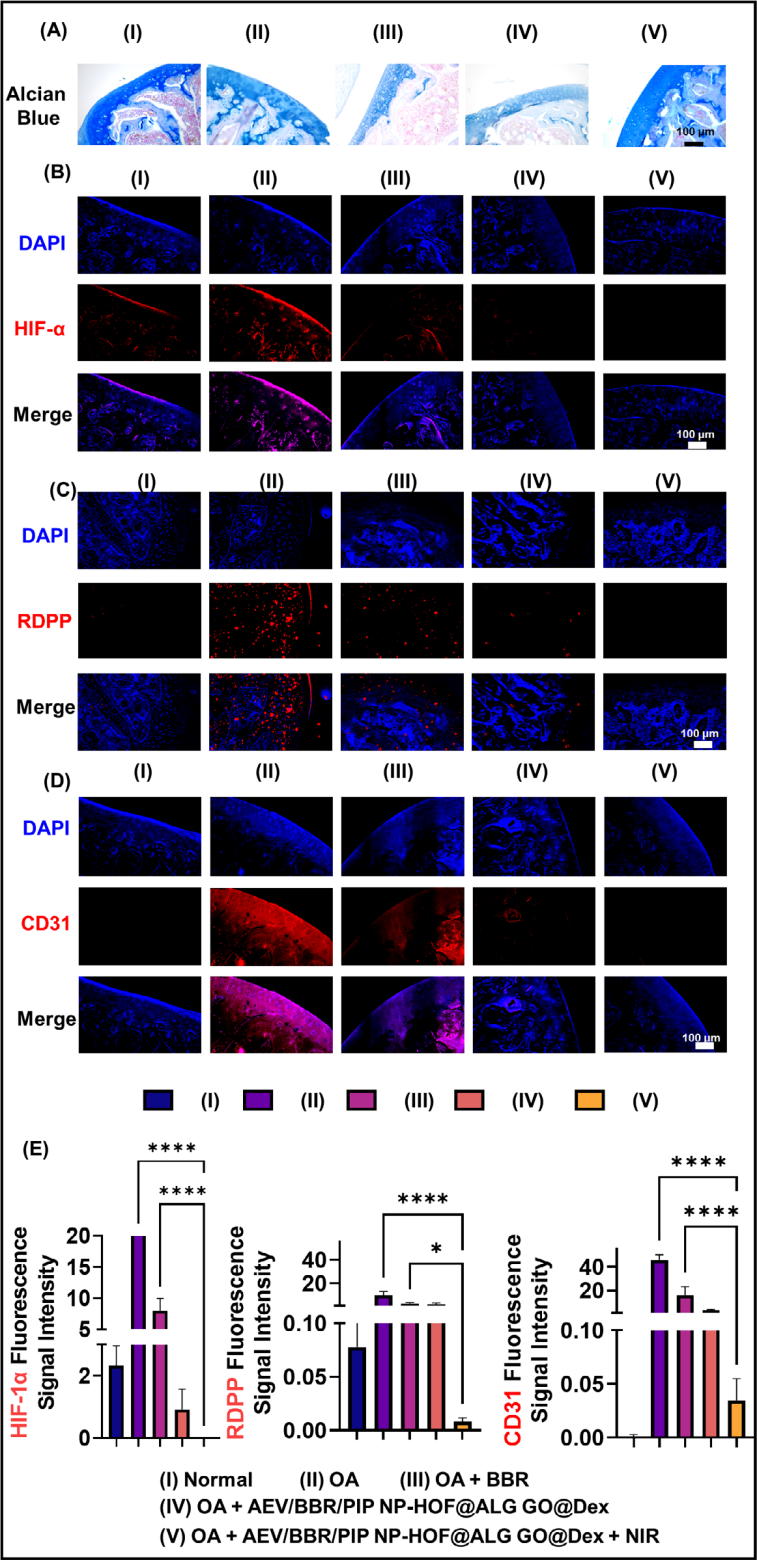
Histological and immunofluorescence analyses of extracellular matrix preservation, hypoxia‐associated signaling, oxygen‐associated status, and angiogenesis in osteoarthritic cartilage. (A) Representative histological images of Alcian blue (AB) staining in different treatment groups, including OA, OA+BBR, OA+AEV/BBR/PIP NP‐HOF@ALG‐GO@Dex, and OA+AEV/BBR/PIP NP‐HOF@ALG‐GO@Dex+NIR, showing cartilage structural changes and extracellular matrix preservation. (B) Representative immunofluorescence images of HIF‐1α staining, showing hypoxia‐associated signaling in cartilage tissue. (C) Representative RDPP staining images, showing oxygen‐associated signals in cartilage tissue across different treatment groups. (D) Representative immunofluorescence images of CD31 staining, showing angiogenesis‐associated signals in cartilage tissue. (E) Quantitative analysis of HIF‐1α, RDPP, and CD31 signals. Compared with the OA, OA+BBR, and OA+AEV/BBR/PIP NP‐HOF@ALG‐GO@Dex groups, the OA+AEV/BBR/PIP NP‐HOF@ALG‐GO@Dex+NIR group showed stronger extracellular matrix preservation, reduced HIF‐1α expression, changed RDPP‐associated oxygen‐related signals, and decreased CD31 expression. Scale bars = 100 µm. Statistical significance was defined as *p* < 0.05 (), *p* < 0.01 ()*, p* < 0.001 (), and *p* < 0.0001 (^****^).

The combined treatment was associated with suppression of HIF‐1α, suggesting attenuation of hypoxia‐associated signaling in the OA cartilage microenvironment. Alcian blue staining (Figure 9A) provided complementary histological evidence consistent with improved extracellular matrix preservation. Notably, the AEV/BBR/PIP NP‐HOF @ALG‐GO@Dex+NIR group exhibited the strongest evidence of extracellular matrix preservation, attenuation of hypoxia‐associated signals, and reduced angiogenic activity, as indicated by Alcian blue staining, HIF‐1α (Figure 9B), RDPP (Figure 9C), and CD31 (Figure 9D) analyses, respectively, compared with the OA, OA+BBR, and OA+AEV/BBR/PIP NP‐HOF@ALG‐GO@Dex groups. Reduced HIF‐1α expression is consistent with attenuation of hypoxia‐associated signaling in the treated OA microenvironment. However, because direct tissue oxygenation was not measured in the present study, this result should not be interpreted as standalone proof of in vivo oxygenation.

To more directly evaluate local oxygen‐associated status in cartilage tissue, RDPP analysis was additionally performed. Compared with the control groups, the treated group showed a higher oxygen‐associated signal (quenching effect), providing tissue‐level evidence consistent with modulation of the local oxygen microenvironment. The addition of cartilage RDPP analysis (Figure 9C) strengthens the interpretation that the treatment may influence local oxygen‐associated conditions in the OA microenvironment. Importantly, this tissue‐level readout provides more direct support than HIF‐1α staining alone, which may also be influenced by broader resolution of inflammatory stress. Quantitative analysis (Figure 9E) revealed that the untreated OA group exhibited severe ECM degradation, reduced hypoxia (as indicated by HIF‐1α expression), and enhanced angiogenesis (as indicated by CD31 expression).

### 2.13 EdU and HSP‐Associated Histological Responses of AEV/BBR/PIP NP‐HOF@ALG‐GO@Dex with NIR Irradiation

This should be accompanied by increased HSP expression and elevated cell proliferation, as indicated by HSP and EdU staining, respectively. Because HSP upregulation may reflect treatment‐associated cellular stress under mild hyperthermia, this finding was interpreted cautiously and not as standalone evidence of cytoprotection.

**FIGURE 10:**
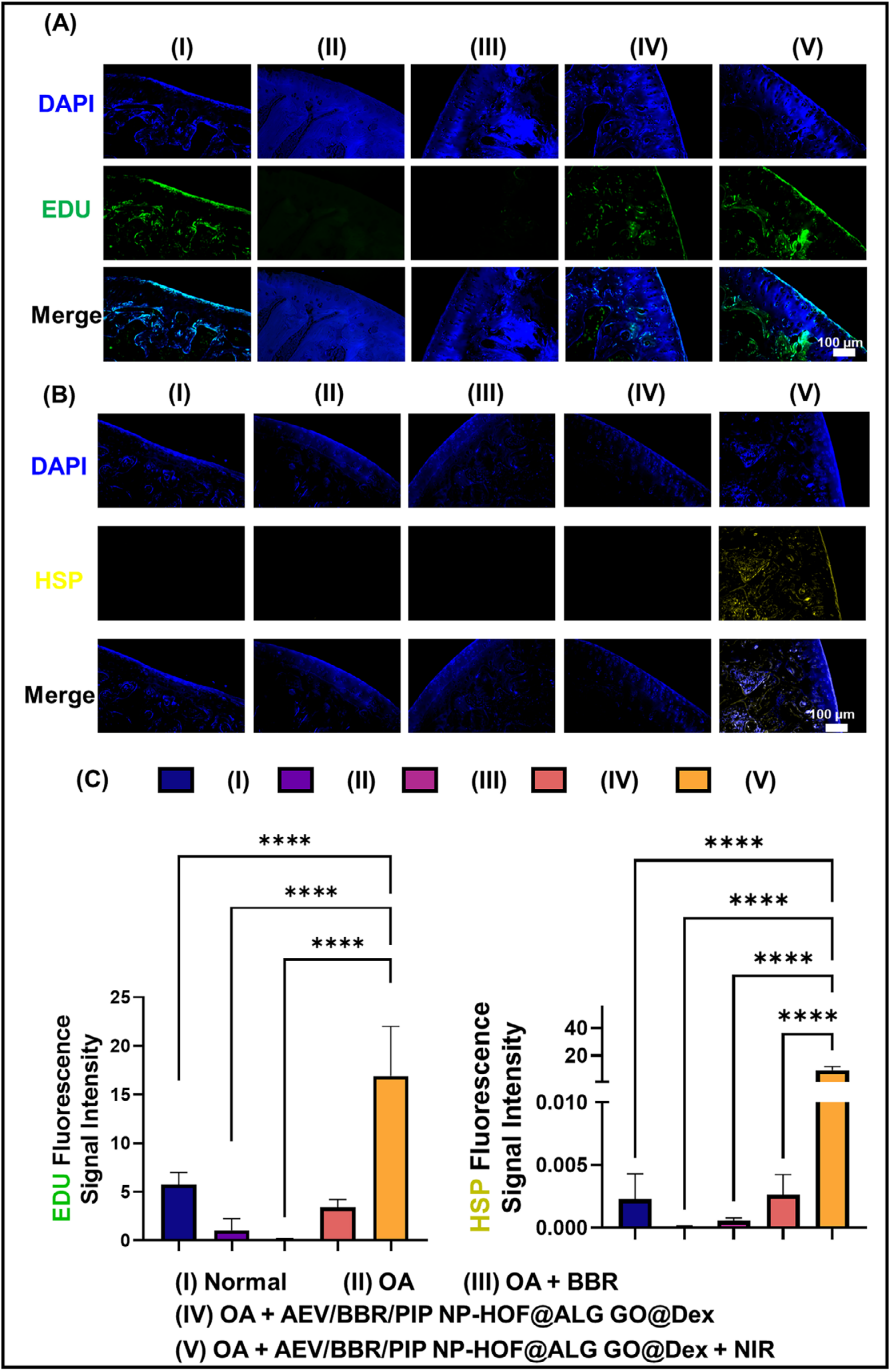
Chondrocyte proliferation and cellular stress response in OA cartilage under different treatment conditions. Representative IF staining of (A) EDU (green) and (B) HSP (yellow) with DAPI nuclear counterstaining (blue) across different groups: OA (untreated), OA+BBR, OA+AEV/BBR/PIP NP‐HOF@ALG‐GO@Dex, and OA+AEV/BBR/PIP NP‐HOF@ALG‐GO@Dex+NIR. The untreated OA group exhibited minimal EDU incorporation and low HSP expression, indicative of impaired chondrocyte proliferation and a lack of an adaptive stress response. Compared with the OA+BBR group, which exhibited relatively low EdU‐associated cellular labeling, the OA+AEV/BBR/PIP NP‐HOF@ALG‐GO@Dex group was associated with increased EdU incorporation and higher HSP expression. Notably, the OA+AEV/BBR/PIP NP‐HOF@ALG‐GO@Dex+NIR group was associated with the strongest EdU and HSP signals, consistent with enhanced proliferation‐ and stress‐associated responses. (C) A quantitative analysis revealed an increase in EDU‐positive cells in the OA+AEV/BBR/PIP NP‐HOF@ALG‐GO@Dex+NIR group compared to the untreated OA group. Scale bars = 100 µm. Statistical significance was defined as *p* < 0.05 (^*^), *p* < 0.01 (^**^), *p* < 0.001 (^***^), and *p* < 0.0001 (^****^).

The potential regenerative and stress‐modulating effects of the nanoplatform were thus further evaluated by EdU and HSP staining, providing complementary information on cell proliferation and stress‐associated responses within the OA cartilage microenvironment. As shown in Figure 10A, the untreated OA group exhibited minimal EDU incorporation, reflecting a severely impaired proliferative capacity in chondrocytes. This observation was further supported by the absence of marked HSP expression, which may reflect limited stress‐response activation under the tested OA conditions. The OA+BBR group also showed a low in the EDU level in cells. However, the limited enhancement of HSP expression (Figure 10B) suggested that BBR alone was insufficient to fully mitigate the oxidative stress burden and inflammatory insults characteristic of OA progression. The OA+AEV/BBR/PIP NP‐HOF@ALG‐GO@Dex group exhibited a further pronounced increase in EDU incorporation, signifying improved chondrocyte proliferation. This increase coincided with a notable elevation in HSP expression, suggesting an altered stress‐associated response in the treated cartilage microenvironment. Notably, the OA+AEV/BBR/PIP NP‐HOF@ALG‐GO@Dex+NIR group exhibited the most pronounced enhancement in both EdU and HSP staining among the evaluated groups. Quantification (Figure 10C) of EdU‐positive cells revealed a multiple‐fold increase relative to the untreated OA group, supporting enhanced proliferation‐associated activity in the treated tissue. Likewise, HSP expression levels were markedly enhanced, indicating a stronger treatment‐associated stress response under the applied conditions. However, this finding should be interpreted cautiously, as increased HSP expression may reflect adaptive heat‐ or treatment‐associated cellular stress rather than direct proof of cytoprotection. The observed improvements are likely associated, at least in part, with the interplay of several interrelated processes. NIR‐responsive stimulation may have contributed to local modulation of the hypoxia‐ and transport‐related microenvironment, while the GO‐containing system may also have supported biological responses relevant to chondrocyte proliferation and survival. Moreover, the presence of AEV‐derived bioactive components provided an additional layer of support by attenuating inflammatory cascades and promoting ECM homeostasis. Because HSP upregulation may reflect adaptive cellular stress under mild hyperthermia, its biological implication in the present study should be interpreted cautiously and not as standalone proof of cytoprotection.d

### 2.14 Systemic Biosafety Evaluation of AEV/BBR/PIP NP‐HOF@ALG‐GO@Dex with and Without NIR Irradiation

**FIGURE 11:**
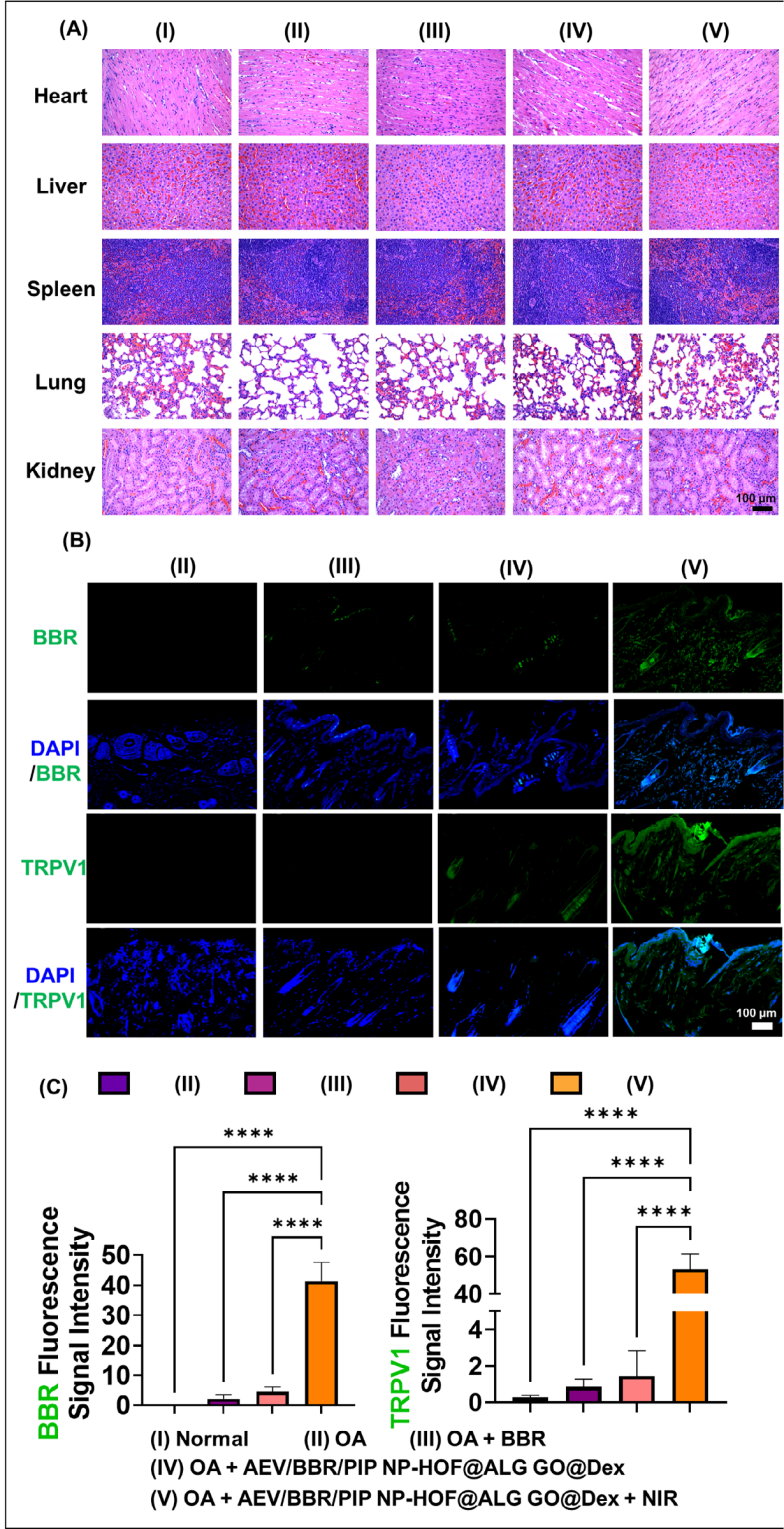
Histological evaluation of major organs to assess the biosafety of AEV/BBR/PIP NP‐HOF@ALG‐GO@Dex with NIR irradiation. (A) Representative H and E‐stained images of the heart, liver, spleen, lungs, and kidneys from different treatment groups, including OA (untreated), OA+BBR, OA+AEV/BBR/PIP NP‐HOF@ALG‐GO@Dex, and OA+AEV/BBR/PIP NP‐HOF@ALG‐GO@Dex+NIR. No overt pathological alterations, inflammatory infiltration, or tissue damage were observed across the examined groups, supporting the biocompatibility of the hydrogel‐based nanoplatform under the tested conditions. These findings provide supportive evidence for its further translational evaluation. (B) Representative fluorescence images showing BBR penetration and TRPV1 expression in different treatment groups, including OA (untreated), OA+BBR, OA+AEV/BBR/PIP NP‐HOF@ALG‐GO@Dex, and OA+AEV/BBR/PIP NP‐HOF@ALG‐GO@Dex+NIR. The OA+AEV/BBR/PIP NP‐HOF@ALG‐GO@Dex+NIR group exhibited the strongest fluorescence signal, suggesting enhanced BBR‐associated tissue penetration and increased TRPV1‐related responses under NIR treatment. (C) Quantitative analysis of BBR‐associated fluorescence intensity and TRPV1 expression in the indicated groups. Scale bars = 100 µm. Statistical significance was defined as *p* < 0.05 (*), *p* < 0.01 (^**^), *p* < 0.001 (^***^), and *p* < 0.0001 (^****^).

A histological analysis (Figure 11A) of major organs, including the heart, liver, spleen, lungs, and kidneys, was performed to evaluate the biosafety of AEV/BBR/PIP NP‐HOF@ALG‐GO@Dex combined with NIR irradiation. H and E staining revealed no detectable pathological alterations or signs of inflammation in any treatment group, affirming the hydrogel's biocompatibility. These findings support the biosafety of the hydrogel under the tested conditions and provide preliminary evidence relevant to its further translational evaluation.

### 2.15 Transdermal Delivery Efficiency and TRPV1‐Dependent Cartilage Targeting Potential

The efficiency of drug delivery through the skin and into deep cartilage lesions was assessed using microscopic imaging (Figure 11B,C). The AEV/BBR/PIP NP‐HOF@ALG‐GO@Dex+NIR group showed the highest level of therapeutic signal penetration among the evaluated groups, exceeding that observed in the untreated OA, BBR‐only, and non‐irradiated hydrogel groups. This enhanced drug delivery may be related to NIR‐responsive effects in the GO‐containing system and associated barrier modulation, potentially involving TRPV1‐related responses that facilitate improved local transport. TRPV1 levels from low to high were in the order of the OA, OA+BBR, OA+AEV/BBR/PIP NP‐HOF@ALG‐GO@Dex, and OA+AEV/BBR/PIP NP‐HOF@ALG‐GO@Dex+NIR groups.

Western blot analysis of tight junction and sensory‐related proteins revealed distinct expression patterns across the OA animals’ treatment groups (Figure S5). Western blot analysis revealed distinct modulation patterns of the tight‐junction protein ZO‐1 and the sensory‐permeability channel TRPV1 across treatment groups. ZO‐1 exhibited the strongest band in the control group (untreated normal animals), indicating a relatively intact epithelial barrier under untreated conditions. In contrast, the three hydrogel‐treated groupsAEV/BBR NP‐HOF@ALG‐GO@Dex, AEV/BBR/PIP NP‐HOF@ALG‐GO@Dex, and AEV/BBR/PIP NP‐HOF@ALG‐GO@Dex + NIRconsistently showed weaker ZO‐1 signals, suggesting a reduction in junctional tightness following hydrogel exposure. This decrease was most evident in the PIP‐or NIR‐induced phototherapy containing groups, aligning with the known ability of TRPV1 activation to transiently reduce tight‐junction integrity and increase paracellular permeability.

Enhanced transdermal delivery was observed for the composited‐containing hydrogel (AEV/BBR/PIP NP‐HOF@ALG‐GO@Dex) under NIR irradiation, as evidenced by improved penetration outcomes in both the ex vivo Franz diffusion assay and the in vivo readouts. Importantly, we interpret the Franz‐cell experiment as a physicochemical permeation assessment under controlled boundary conditions (barrier‐limited transport across skin), rather than as evidence of ion‐channel activation in ex vivo skin. In this context, the increased permeation in the NIR group is consistent with passive enhancement driven by mild hyperthermia at the skin surface (e.g., increased diffusivity, altered interfacial transport resistance, and formulation–skin contact dynamics) together with permeation‐facilitating effects of piperine as a well‐known penetration modulator [28]. Potential relevance to TRPV1 and ZO‐1 regulation was supported by analyses of viable in vivo treated tissues collected from the irradiated region. Specifically, Western blotting and immunofluorescence showed a treatment‐dependent increase in TRPV1 expression and a concomitant reduction in ZO‐1 in the PIP‐containing groups, with the most pronounced change under the PIP + NIR condition. This pattern is consistent with prior reports indicating that TRPV1‐related signaling may be associated with transient changes in tight‐junction organization and paracellular permeability. In this context, the present findings support a two‐level interpretation: (i) the ex vivo Franz diffusion results indicate that the formulation, together with mild NIR‐controlled hyperthermia, can enhance transdermal transport under standardized conditions; and (ii) the in vivo changes in TRPV1 and ZO‐1 expression provide supportive biological context for barrier‐associated modulation in living skin, consistent with the strongest penetration‐associated signal observed in the PIP + NIR group. Overall, these findings might support the plausibility of the proposed transdermal delivery strategy while maintaining an appropriately bounded mechanistic interpretation.

In this context, the delivery enhancement under in vivo irradiation should be viewed as a multicomponent outcome rather than a heat‐only effect. NIR exposure concurrently accelerated formulation‐level drug liberation from the hydrogel depot (consistent with the NIR‐responsive release behavior) and, together with the penetration‐enhancing property of piperine, increased net transdermal transport when the barrier resistance was transiently lowered by mild hyperthermia. Therefore, the observed improvement did not require a long dwell time at ≥ 40°C; instead, it arose from the combined contributions of transient barrier loosening and NIR‐triggered release/partitioning at the formulation level.

## 3 Conclusions

This study sets forth the development and assessment of an innovative multimodal nanomedicine platform, AEV/BBR/PIP NP‐HOF@ALG‐GO@Dex, for the thorough management of OA. The platform efficiently tackles critical pathological processes in OA by integrating the integrated capabilities of AEV, BBR, PIP, and GO into a biocompatible polysaccharide gel matrix, addressing inflammation, hypoxia, oxidative stress, immunological regulation, and pain driven by angiogenesis.

Under NIR irradiation, the GO‐containing component may contribute to light‐responsive local effects associated with enhanced transdermal transport, a shift in macrophage polarization toward an anti‐inflammatory phenotype, and reduction of hypoxia‐ and oxidative stress–associated features. The bioactive characteristics of AEV, BBR, and PIP may have further contributed to the observed benefits, particularly with respect to inflammation‐associated modulation and tissue recovery–related responses.

Taken together, the present data support an association between NIR‐responsive physicochemical behavior in the GO‐containing formulation and the downstream biological responses observed in this study, including oxidative stress attenuation, macrophage polarization shift, and modulation of hypoxia‐associated signals. However, these findings should be interpreted as supportive rather than as definitive proof of direct causal molecular pathways.

Similarly, although reduced HIF‐1α staining was observed in vivo, this finding may also reflect broader resolution of inflammatory stress rather than direct tissue oxygenation alone. Direct oxygenation measurements will therefore be necessary in future studies to clarify this mechanism. Although the newly added RDPP analysis provides more direct tissue‐level evidence relevant to local oxygen‐associated status, further studies using complementary oxygenation measurements will still be valuable for fully resolving the precise mechanism of hypoxia modulation in vivo.

The established system was associated with reduced knee inflammation, as indicated by thermal imaging, and improved joint function, as supported by gait analysis and MRI evaluation. Overall, this study supports the potential of combining advanced nanomedicine engineering, transdermal administration, and phototherapy as a noninvasive strategy for multifaceted osteoarthritis management, with possible broader relevance to inflammation‐associated degenerative disorders.

Although surface thermal imaging confirmed localized heating at the treatment site, the present data do not establish direct mild hyperthermia within deep joint tissues; therefore, the observed in vivo benefits are interpreted more conservatively in terms of surface‐mediated transport enhancement and associated downstream biological responses.

Although the added comparative data support a stabilizing and release‐modulating role of HOF incorporation, direct profiling of potential HOF‐derived leachates or degradation products was not performed in the present study and warrants future investigation.

Through the combination of engineered AEV‐based nanocargo and an NIR‐responsive hydrogel matrix, this work establishes a noninvasive therapeutic platform for local modulation of the OA microenvironment. At the same time, several mechanistic aspects remain to be further elucidated. Although the current data support NIR‐associated redox modulation in the GO‐containing system, the PB assay, ORP measurements, and dissolved oxygen analysis alone do not provide definitive evidence for a complete photocatalytic water‐splitting process under physiological conditions. Therefore, the corresponding mechanistic interpretations should be viewed as supportive rather than fully resolved. Future investigations should further optimize treatment parameters, assess long‐term safety and therapeutic durability, and incorporate more direct physicochemical and molecular validations to define the operative pathways and facilitate future clinical translation for OA and related disorders.

## 4 Materials and Methods

### 4.1 Materials

BBR, PIP, GO, ALG, and Dex were purchased from Sigma–Aldrich (St. Louis, MO, USA). Additional biochemical reagents were obtained from Thermo Fisher Scientific (Cleveland, OH, USA) and were all of high‐performance liquid chromatographic (HPLC) grade.

AEVs were isolated using a combination of cellular ultrasonication and filtration techniques, as described in a previous study [18]. Fresh *Malus domestica* (apple) for AEV preparation was sourced from Chuan Lian Enterprise (Taipei, Taiwan). To isolate AEVs, the apples were processed to extract vesicles using ultrasonication at 40% power in a water bath for 30 min. The resulting suspension was filtered through a sterile syringe filter with a 0.22‐µm pore size to obtain purified AEVs for subsequent experimental applications. All materials and reagents were handled and stored under conditions recommended by the respective manufacturers to ensure their stability and integrity during the study.

### 4.2 Formulation Development and Physicochemical Characterization

0.05 M trimesic acid and 0.1 M imidazole were separately dissolved in a 10% DMSO solution and heated to approximately 90 °C. The two solutions were then mixed and stirred for 30 min to facilitate the formation of the HOF. Upon completion of the reaction, the mixture was allowed to stand and gradually cooled to a low temperature, promoting the precipitation of the HOF particles. The resulting precipitate was washed with distilled water to remove residual solvent and unreacted reagents. Finally, the purified nanoparticles were dried under vacuum to remove any remaining water and solvent. The optimized formulation was prepared by combining a fixed concentration of AEVs (3.8 × 10^8^ cells/mL), BBR (1 mg/mL), and PIP (1 mg/mL) with various weight ratios of Pro and HOFs (6.25/0, 15.8/0, 15.8/5.5, 20/0, and 24/0 w/w). The assembly of AEV/BBR/PIP NP‐HOFs and GO was evaluated by multiple physicochemical characterization methods, including dynamic light scattering (DLS), nanoparticle tracking analysis (NTA), and transmission electron microscopy (TEM). Particle sizes and zeta potentials were evaluated using DLS and the NTA, while the loading efficiency of BBR was determined via spectrometric assays.A 2,2‐diphenyl‐1‐picrylhydrazyl (DPPH) inhibition assay was conducted to evaluate the antioxidant activity [29].

The photothermal effects of the samples were tested upon near‐infrared (NIR) irradiation (808 nm, 1.5 W/cm^2^) for 5 min. Photochemical responsiveness was measured by conducting a Prussian blue spectrometric examination following identical NIR irradiation circumstances [30]. The extent of oxygen reduction was assessed using oxidation‐reduction potential (ORP) technology after NIR treatment (808 nm, 1.5 W/cm^2^, 5 min). Additionally, dissolved oxygen changes in the system were measured using an oxygen meter to assess oxygen‐related responses under the tested conditions.

The synthesized AEV/BBR/PIP NP‐HOFs and GO were then incorporated into a hybrid hydrogel matrix consisting of Dex (10 mg) and ALG (10 mg) via a coordination technique. Specifically, AEV/BBR/PIP NP‐HOFs (1 mg) and GO (0.5 mg) were dispersed in ultrapure water alongside Dex and ALG under continuous stirring, resulting in the formation of AEV/BBR/PIP NP‐HOF@ALG‐GO@Dex. Hydrogel samples were concentrated through centrifugation and consequently dispersed in a phosphate‐buffered saline (PBS) solution for further applications.

The mechanical and structural features of AEV/BBR/PIP NP‐HOF@ALG‐GO@Dex were characterized using texture analysis (TA), rheometry, and scanning electron microscopy (SEM). These analyses supported the incorporation of the nanostructured components within the hydrogel matrix and provided structural evidence relevant to its subsequent functional evaluation.

Ex vivo transdermal permeation was evaluated using vertical Franz diffusion cells (effective diffusion area: 0.785 cm^2^; receptor volume: 5 mL). Full‐thickness rat skin was harvested immediately after sacrifice, and subcutaneous fat was carefully removed. The skin was equilibrated in phosphate‐buffered saline (PBS) at room temperature for 10 min before mounting. The skin was clamped between the donor and receptor chambers with the stratum corneum facing the donor compartment, and the receptor chamber was filled with PBS (pH 7.4) to maintain sink conditions for BBR.Baseline temperature of the diffusion cells was maintained at 30 ± 0.5°C (or the specified physiological skin surface temperature).

For dosing, a formulation volume corresponding to 1.0 mL per cell (total BBR loaded: 48 µg) was applied uniformly onto the donor side within the diffusion area. For the NIR group, the donor surface was irradiated with an 808‐nm NIR laser (2 W cm^−^^2^) for 5 min. During irradiation, the skin surface temperature was recorded in real time and controlled to remain within the mild‐hyperthermia window (∼42°C) by adjusting the irradiation distance and/or applying intermittent irradiation if needed. The non‐irradiated group used an identical cell setup and temperature control without laser exposure.

At predetermined time points (e.g., 0.25, 0.5, 1, 5, 10, 30 min), aliquots (200 µL) were withdrawn from the receptor compartment and immediately replaced with an equal volume of fresh pre‐warmed receptor medium to maintain constant volume and sink conditions. The permeated BBR was quantified by fluorescence using a calibrated standard curve measured under identical conditions. Data were expressed as cumulative permeation normalized to dose, reported as percentage of the applied BBR dose (%), calculated as (cumulative amount of BBR permeated into the receptor compartment, with appropriate correction for serial sampling and replenishment) divided by the initially applied BBR amount, multiplied by 100. Each condition was performed with n = 3 independent skin samples, and data were presented as mean ± SD.

### 4.3 In Vitro Cell‐Based Biological Assessments

RAW macrophage cells were obtained from ATCC (Manassas, VA, USA) and cultured in α‐minimum essential medium (α‐MEM) supplemented with 100 U/mL penicillin‐streptomycin (1% P/S) and 10% fetal bovine serum (FBS; Gibco, Grand Island, NY, USA). Cells were cultured with 5% CO_2_ at 37°C in a humidified incubator. Cell surface protein markers were analyzed using fluorescence microscopy with monoclonal antibodies sourced from Thermo Fisher Scientific.

Chondrocytes were purified from articular cartilage of male Wistar rats (approximately 10 weeks old), supplied by Biolasco (Taipei, Taiwan), in accordance with a previously established protocol. Briefly, cartilage was diced into small fragments and enzymatically digested with 0.2% type II collagenase (Sigma, St. Louis, MO, USA) in Dulbecco's modified Eagle medium (DMEM)/F12 medium (Invitrogen, Carlsbad, CA, USA.) at 37°C for 6 h. The resultant cell suspension was filtered to isolate chondrocytes, which were cultured in DMEM/F12 medium supplemented with 10% FBS and 1% P/S. The medium was replenished every other day to maintain optimal cell viability. Chondrocytes were used within three passages to avoid phenotypic drift or dedifferentiation.

To mimic inflammatory conditions, RAW cells and chondrocytes were pretreated with lipopolysaccharide (LPS) at a concentration of 1 µg/mL for 24 h under standard culture conditions (37°C, 5% CO_2_). Following treatment with the experimental formulations, oxidative stress levels and immunomodulatory responses were evaluated. An Amplex red assay was employed to quantify oxidative stress, while cluster of differentiation 206 (CD206) and CD86 immunofluorescence (IF) markers were used to assess macrophage polarization. Fluorescence signals were visualized using fluorescence microscopy and quantified using ImageJ software (National Institutes of Health, Bethesda, MD, USA), enabling a detailed analysis of cellular responses. These assays provided additional evidence relevant to the antioxidant‐ and anti‐inflammatory–associated activity of the tested formulations.

### 4.4 In Vivo Experimental Validation Using Animal (Rat) Models

Ten‐week‐old male Wistar rats, weighing 200–300 g, were obtained from Biolasco for this investigation. The experimental procedures were conducted in compliance with the approved protocol under TMU IACUC approval no. SHLAC2024‐0143 a. Animals were housed under controlled conditions at a temperature of 25°C and 50% humidity. A knee joint (TMJ) OA model was established by injecting monosodium iodoacetate (MIA) into the TMJ, as described in a previous study [31, 32]. Specifically, 20 mg/mL of MIA (Sigma–Aldrich) was dissolved in 50 µL PBS and bilaterally injected into the anterosuperior compartments of the TMJs.

Following 3 weeks of OA induction, rats were randomly assigned to an experimental group: OA (control), OA+BBR, OA+AEV/BBR/PIP NP‐HOF@ALG‐GO@Dex (topical administration), and OA+AEV/BBR/PIP NP‐HOF@ALG‐GO@Dex (topical administration) with NIR irradiation. The hydrogel patch was applied every two days for three weeks, with NIR irradiation performed under identical conditions. The gel matrix was detached from the skin by washing with PBS.

An IR thermal camera (MET‐FLTG300+2 NIR, S.E.A.T, Kaohsiung City, Taiwan) was used to monitor in vivo thermal changes during treatment. The biodistribution of therapeutic agents was assessed using an in vivo imaging system (IVIS), and magnetic resonance imaging (MRI) scans were performed with a PharmaScan 7.0 T system (BioSpin MRI, Bruker, Germany). Gait analysis and water maze experiments were conducted to evaluate arthritis severity and functional recovery after treatment. For MRI grayscale/color‐scale quantification, a predefined region of interest (ROI) was manually outlined over the knee joint lesion area on T2‐weighted images using the same anatomical landmarks across all animals. Image analysis was performed in a blinded manner, with the evaluator masked to treatment allocation during ROI selection and signal extraction. Mean grayscale/color‐scale intensity within each ROI was measured and normalized to the corresponding background/reference region in the same image to reduce inter‐scan variation. The normalized values were then used for statistical comparison among groups. Image processing and grayscale/color‐scale quantification were performed using ImageJ.

Four weeks post‐treatment, all animals were euthanized via CO_2_ inhalation for tissue collection and analysis. Knee tissues were excised, embedded, and sectioned for a histological examination. Sections were stained using IF, and Alcian blue (AB, Sigma) to assess acid proteoglycan levels in cartilage. Additional staining included markers for macrophage polarization (CD206 for M2 macrophages and CD86 for M1 macrophages, both 1:100, Abcam), hypoxia (HIF‐1α), oxygen levels or hypoxic status (RDPP) angiogenesis (CD31/VEGF), proliferation (5‐ethynyl‐2'‐deoxyuridine, EDU) assay kits, inflammation (IL‐6), and heat shock protein (HSP) expression (HSP70, 3:1000, Abcam), TRPV1 staining for skin. ROS levels were measured utilizing an Amplex red assessment.

To evaluate potential systemic toxicity, tissues from the liver, heart, kidneys, lungs, and spleen were collected, sectioned, and examined by H and E staining. Each experimental group included three animals. These analyses provided histological evidence relevant to the systemic safety assessment of the tested formulations.

ZO‐1 and TRPV‐1 protein expression was analyzed by Western blotting. Following treatment, cells were washed with PBS and lysed in RIPA buffer containing protease inhibitors. After centrifugation, protein concentrations were quantified using a BCA assay. Equal amounts of protein were separated by SDS‐PAGE, transferred to PVDF membranes, and blocked with 5% non‐fat milk. Membranes were incubated overnight at 4°C with primary antibodies against ZO‐1, TRPV‐1, and GAPDH, followed by HRP‐conjugated secondary antibodies. Protein bands were detected using enhanced chemiluminescence, with GAPDH serving as the loading control.

### 4.5 Statistical Analysis

Quantitative data are presented as the mean ± standard deviation (SD). Statistical differences among groups were analyzed using Graphpad Prism (USA). Statistical significance was defined as *p* < 0.05 (^*^), *p* < 0.01 (^**^), *p* < 0.001 (^***^), and *p* < 0.0001 (^****^).

## Author Contributions

The manuscript was written through the contributions of all authors. All authors approved the final version of the manuscript.

## Ethics Statement

This study was approved under guidelines of the Animal Ethics and Use Committee of Taipei Medical University.

## Consent

All of the authors involved in this study agreed to its publication.

## Conflicts of Interest

The authors declare no conflict of interest.

## Supporting information




**Supporting File**: adhm71330‐sup‐0001‐SuppMat.docx.

## Data Availability

The data that support the findings of this study are available from the corresponding author upon reasonable request.
