# Supplementary material for: HOF‐Stabilized Apple EV Nanocargo in an NIR‐Responsive Hydrogel Enables On‐Demand Therapeutic Modulation for Transdermal Deep‐Joint Osteoarthritis Therapy
Source: Adv Healthc Mater. 2026 Jul 15;15(29):e71330. doi: 10.1002/adhm.71330 (PMC13447883; doi:10.1002/adhm.71330)
Supplement: Supplementary file 1 [file ADHM-15-0-s001.docx]

***Supplementary Information***

**Figure S1.** **Photoresponsiveness and oxygen-related characterization of GO under NIR irradiation, together with ^1H NMR spectra of the composite formulation and its individual components.** (A) Representative photoactivation evaluation of GO under NIR irradiation, showing the photoinduced potential response measured using an electrochemical workstation. (B) Photothermal performance of GO under NIR irradiation, including the temperature elevation profile and representative infrared thermometer images recorded during laser exposure.(C) Dissolved oxygen analysis of GO under NIR irradiation, showing changes in oxygen-related behavior measured using a dissolved oxygen meter under repeated NIR stimulation.(D)^1H NMR spectra of the AEV/BBR/PIP NP-HOF@ALG-GO@Dex composite and its individual components.

**Figure S2.** Franz diffusion cell analysis of BBR release at the 30-min time point. Free BBR showed baseline fluorescence intensity, while incorporation into AEV/BBR/PIP NP-HOF@ALG-GO@Dex resulted in a modest increase in release. Significantly enhanced BBR diffusion was observed from AEV/BBR/PIP NP-HOF@ALG-GO@Dex, with the highest release achieved under NIR irradiation, indicating the combined effects of PIP incorporation and NIR stimulation on transdermal BBR delivery.

**Figure S3. Evaluation of the hypoxia-relieving and immunomodulatory effects of GO under NIR irradiation in RAW cells under a nitrogen-purged hypoxic condition.** Schematic images showing the establishment of the hypoxic environment by nitrogen purging are presented at the top. Representative immunofluorescence images show intracellular ROS levels (DCFDA, green), macrophage polarization markers (CD86, red; CD206, green), and HIF-1α expression (red) in RAW cells under different treatment conditions. DAPI (blue) was used for nuclear staining. Compared with the hypoxic control group, the GO+NIR-treated group exhibited reduced ROS accumulation, attenuated HIF-1α expression, and a shift in macrophage polarization from a pro-inflammatory phenotype toward a more reparative phenotype, as indicated by decreased CD86 and increased CD206 signals. These findings support that GO under NIR irradiation may help alleviate hypoxia-associated cellular stress and inflammatory responses under the tested conditions. Scale bar = 100 μm.

**Figure S4.** Continuous skin/joint-surface temperature monitoring of the irradiated knee during light exposure. Temperature at the knee surface was recorded in real time throughout irradiation to generate full temperature–time profiles (complementing endpoint infrared thermograms). For animals, the measurement position was fixed at the center of the irradiated knee region to ensure consistent placement and direct comparability across groups.

**Figure S5.** Western blot analysis of epithelial barrier and sensory-permeability proteins following OA animals’ hydrogel treatments. Representative immunoblots of ZO-1 and TRPV1 in skin (irradiated region) collected from the indicated groups: Control (normal untreated animals), AEV/BBR NP-HOF@ALG-GO@Dex, AEV/BBR/PIP NP-HOF@ALG-GO@Dex, and AEV/BBR/PIP NP-HOF@ALG-GO@Dex + NIR.
